# Comparative Transcriptomics as a Key to Understanding the Adaptation Mechanisms of Baikal Sculpins to the Deep-Water Habitat

**DOI:** 10.3390/biology14121762

**Published:** 2025-12-09

**Authors:** Yulia P. Sapozhnikova, Anastasiya G. Koroleva, Tuyana V. Sidorova, Evgenia A. Vakhteeva, Alexander A. Epifantsev, Sergey A. Potapov, Vera M. Yakhnenko, Lyubov V. Sukhanova, Sergei V. Kirilchik, Tatyana V. Butina, Ivan A. Nebesnykh, Igor V. Khanaev

**Affiliations:** Limnological Institute, Siberian Branch of the Russian Academy of Sciences, 3 Ulan-Batorskaya, Irkutsk 664033, Russia; ankor-2015@yandex.ru (A.G.K.); tuyana_be@mail.ru (T.V.S.); vakhteevaevgenia@mail.ru (E.A.V.); epifantsevalexander@yandex.ru (A.A.E.); poet1988@list.ru (S.A.P.); vera@lin.irk.ru (V.M.Y.); lsukhanova@yandex.ru (L.V.S.); kir@lin.irk.ru (S.V.K.); tvbutina@mail.ru (T.V.B.); canis-87@mail.ru (I.A.N.); igkhan@lin.irk.ru (I.V.K.)

**Keywords:** Lake Baikal, adaptation, deep-water habitat, sculpins, Cottoidei, transcriptome, differential gene expression

## Abstract

Understanding the molecular mechanisms that enable vertebrate adaptation to deep-water environments remains a central goal in evolutionary biology. This study used comparative transcriptomics of skeletal muscle tissue to investigate these mechanisms in four endemic sculpin fish species (Cottoidei) from Lake Baikal, which inhabit different depth niches ranging from pelagic to benthic-abyssal zones up to 1642 m. Pelagic species showed increased activity in genes involved in sarcomere structure, calcium handling, and motor proteins, indicating adaptations for sustained locomotion. In contrast, deep-benthic specialists showed enrichment in pathways for glycolytic metabolism, proteasome function, and ubiquitination, reflecting adaptations for energy efficiency and protein homeostasis in a high-pressure environment. We conclude that the colonization of the Baikal abyssal zone by sculpins relies on a suite of shared molecular mechanisms, with distinct ecological pressures driving specific transcriptional changes in motility, metabolic strategy, and cellular integrity. This study provides a systems-level model for deep-water adaptation in vertebrates.

## Simple Summary

The deep-water environments of lakes and oceans, characterized by perpetual darkness and high pressure, present extreme challenges for animal life. Understanding how organisms genetically adapt to these conditions is a fundamental goal in evolutionary biology. Lake Baikal provides a unique natural laboratory, as its endemic sculpin fish (Cottoidei) have diversified to occupy all depth zones, including the deep abyssal zone (up to 1642 m). Our research investigated the genetic mechanisms underlying this adaptation by comparing pelagic open-water species with benthic (demersal) abyssal dwellers. We found that pelagic sculpins use genes suited for sustained swimming. In contrast, benthic sculpins activate a specific set of genes related to efficient energy use, cellular maintenance, and structural integrity, which are crucial for conserving energy and withstanding high pressure. These findings reveal a core set of genetic principles for survival in deep freshwater habitats. This knowledge is crucial for conserving the unique biodiversity of ancient ecosystems like Lake Baikal, especially as they face increasing pressures from climate change and human activity.

## 1. Introduction

Lake Baikal, a UNESCO World Heritage Site, serves as a natural freshwater laboratory for evolutionary biology and comparative genomics. Its ancient origin, tectonic history, and unique hydrological conditions have fostered an extraordinary endemic fauna. In particular, its fish assemblage represents a remarkable model for studying adaptive radiation and speciation [1,2]. Among the most diverse and ecologically significant groups, which make up to 80% of the lake’s total fish biomass, are the sculpins of the group Cottoidei [3]. Baikal sculpins are a phylogenetically closely related group that radiated from a common marine ancestor within the last 2–2.5 million years [1,4]. This radiation has produced about 40 endemic species that occupy a wide range of ecological niches [5]. This diversification is especially evident along the bathymetric gradient, with species adapted to specific depths ranging from shallow waters to the abyssal zone (up to 1642 m). The evolutionary history and ecology of these fish are well-documented, providing a solid foundation for comparative studies [2,3,4,5,6,7,8,9,10,11,12,13,14,15]. Understanding the mechanisms underlying their ecological adaptations is crucial for revealing the evolutionary processes that shape biodiversity in such isolated ecosystems.

The deep-water habitat of Lake Baikal presents formidable physiological challenges for aquatic life. These include low temperatures consistently near 4 °C, high hydrostatic pressure (over 160 atm) and perpetual darkness [15]. However, a key factor that distinguishes Baikal from other deep lakes and enables its rich deep-water fauna is the persistent presence of dissolved oxygen throughout the entire water column. Oxygen concentrations are high (9–14 mg/dm^3^) and, critically, oxygen saturation in the bottom waters rarely falls below 70%, due to unique seasonal deep-water renewal mechanisms [16]. This oxygen-rich environment mitigates a challenge that typically constrains life in the deep zones of most freshwater ecosystems. Nevertheless, for vertebrates, high hydrostatic pressure remains a pervasive stressor that can disrupt protein folding, membrane fluidity, and the function of neuronal and contractile tissues, posing a significant barrier to colonization [17,18,19]. Thus, how Baikal sculpins have overcome these constraints to thrive in deep waters is a central question in evolutionary physiology.

In the post-genomic era, transcriptomics has emerged as a powerful tool for understanding the molecular basis of phenotypic adaptation. By quantifying gene expression levels across tissues and conditions, transcriptomic analyses can identify key metabolic pathways, stress responses, and structural modifications critical for environmental adaptation [20,21]. Comparative transcriptomics, which compares expression profiles between closely related species from different habitats, is particularly effective. This approach allows for the identification of conserved core molecular functions and those altered by natural selection to cope with specific environmental pressures [20,22]. Such studies have successfully revealed adaptive mechanisms in other deep-water organisms, showing changes in genes related to energy metabolism and apoptosis in response to high pressure [22,23,24,25,26]. While adaptations in marine deep-sea fish are increasingly studied, systematic investigations of shared molecular principles in freshwater deep-water ecosystems remain scarce. To our knowledge, this is the first comparative transcriptomic study of endemic freshwater sculpins (Cottoidei, Scorpaeniformes) inhabiting contrasting deep-water niches (pelagic versus benthic-abyssal). Additionally, it provides the first transcriptomic data for the deep-water freshwater Cottoidei. To address this gap and provide a comprehensive, systems-level model for adaptation in a unique ancient deep-water ecosystem, we developed a comparative framework that includes two species from each ecological group: pelagic and benthic-abyssal.

In this study, we apply a comparative transcriptomic framework to investigate the molecular adaptations of endemic Baikal sculpin species from two different genera: the benthic (demersal) abyssal *Batrachocottus* (or *Adipocottus*, according to [5]) and the pelagic *Comephorus*, which inhabits the water column. The selection of these species is based on their distinct and well-documented bathymetric distributions and ecological strategies, providing a strong comparative framework for studying depth-related adaptations. The two benthic-abyssal sculpins, the fatty sculpin *Batrachocottus* (*Adipocottus*) *nikolskii* (Berg, 1900) and the spotty-fins sculpin *Batrachocottus* (*Adipocottus*) *multiradiatus* (Berg, 1907), inhabit muddy bottoms at depths of 200 to 1400 m and 15–20 to 950 m, respectively, with *B. multiradiatus* being more eurybathic than *B. nikolskii* [3,5,6,27]. In contrast, the two pelagic species, the big golomyanka *Comephorus baicalensis* (Pallas, 1776) and the small golomyanka *Comephorus dybowski* (Korotneff, 1905), are secondary pelagic fishes, characterized by viviparity and a lack of association with the bottom, inhabiting the entire water column of the open lake down to maximum depths of 1642 m [3,6,15], although individual *Comephorus* specimens touching soft sediments have been noted previously [28]. These species, which lack a swim bladder, perform extensive vertical migrations and do not form schools [3,7,8]. Unlike typical marine pelagic fish, *Comephorus* species are not highly active swimmers. Instead, they hover in the water column, maintained by neutral buoyancy and their elongated, broad pectoral fins [3,29]. However, significant activity during feeding has also been observed in these species, indicating they can be in continuous motion [15]. This phylogenetic context, comparing closely related sculpin species that have diverged to occupy contrasting deep-water niches (benthic-abyssal versus pelagic), allows for a focused investigation into the molecular mechanisms underlying adaptive specialization.

In this work, we focus on skeletal muscle as a key tissue. This tissue was selected because it is the dominant tissue in fish, comprising a major portion of body mass, and plays a vital role in metabolic balance and serves as the primary motor for swimming, a fundamental and defining activity of these vertebrates [20,30]. Furthermore, as a key metabolic organ, skeletal muscle provides critical insights into adaptations for energy efficiency and proteostasis under high hydrostatic pressure [20,22,30], making it an ideal system to uncover the molecular mechanisms underlying ecological specialization. From a practical standpoint, skeletal muscle is an easily accessible tissue that allows rapid sampling from deep-water, wild-caught specimens, which was critical for minimizing RNA degradation and ensuring high-quality data. While studying other tissues would provide a more comprehensive view, skeletal muscle offers a powerful and rational starting point [20,22,30].

For the first time, we describe and compare the transcriptional profiles of skeletal muscle in these species, focusing on identifying the genetic signatures associated with a deep-water lifestyle. We hypothesize that deep-water benthic (demersal) sculpins will show distinct expression patterns in genes involved in pressure sensing, membrane composition, metabolic reprogramming, and cellular stress response compared to pelagic species. This research aims to provide a deeper, systems-level understanding of the molecular mechanisms that underpin the successful adaptation of vertebrate life to the abyssal environment of Lake Baikal.

## 2. Materials and Methods

### 2.1. Field Sampling and Trawl Operations

**Figure 1:**
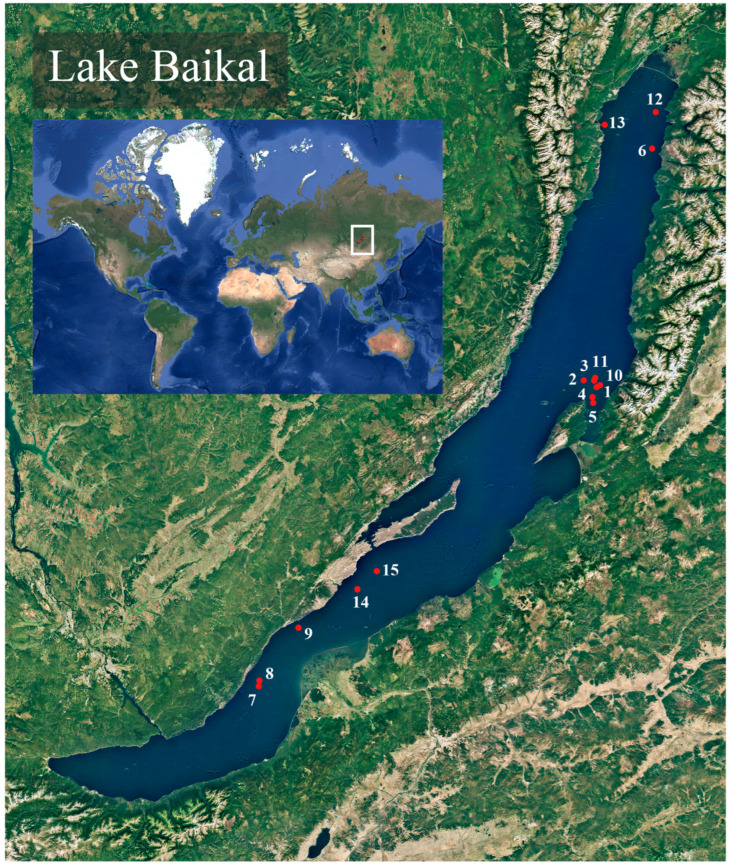
Map of trawling locations on Lake Baikal. Red dots represent trawling start points, corresponding to the trawl numbers in Table 1. This map was created using QGIS v.3.40.12.

Specimens of deep-water endemic sculpins (Cottoidei) were collected during a research expedition aboard the research vessel G. Yu. Vereshchagin (operated by the Center for Collective Use «Research vessels Center of LIN SB RAS on Lake Baikal»), managed by the Limnological Institute of the Russian Academy of Sciences, in August 2023 and 2024. Sampling was conducted in the central and northern basins of Lake Baikal, specifically at depths ranging from 218 to 1397 m (Table 1, Figure 1).

A beam trawl, equipped with a fine-mesh cod-end liner (10 mm mesh) to minimize physical damage to the specimens, was deployed as the primary sampling gear. To reduce the duration of stress and prevent degradation of tissue quality, trawl hauls were designed to be short, typically lasting 40–60 min at the target depth. Upon retrieval, the trawl was immediately brought on deck, and the cod-end contents were carefully emptied into a large, insulated tank continuously supplied with fresh, chilled lake water.

Baikal sculpin species were identified in the field before tissue sampling based on macroscopic morphology, guided by published keys and assisted by I.V. Khanaev. Only live, actively respiring, and morphologically intact individuals of the target species were selected for sampling. To exclude the influence of major ontogenetic shifts in gene expression, sampling was limited to sexually mature adult representatives of each species. Selected specimens were rapidly euthanized by incising the spinal cord, a method that minimizes the physiological stress response. Within a critical window of 60–90 s post-euthanasia, the target tissue (skeletal muscle) was dissected. Dissections were performed using sterile, RNase-free forceps and scalpels.

Immediately upon dissection, approximately 20–30 mg of each tissue sample was placed into a pre-labeled, pre-chilled 0.6 mL cryovial containing 0.3 mL of Reagent for ExtractRNA (BC032, an analog of TRIzol, Evrogen, Moscow, Russia). TRIzol, a monophasic solution of guanidine isothiocyanate and phenol, acts as a powerful denaturant, instantly inactivating RNases and preserving the RNA’s integrity at the moment of fixation. A total of 84 specimens (18–23 fish per species) were sampled (Table 2).

The Ethics Committee of the Limnological Institute SB RAS accepted the experiments and the publication of the results in the press in accordance with Russian laws, standards and guidelines on animal welfare (Protocol #3, 1 November 2025).

### 2.2. RNA Extraction and Subsequent Preparation

Total RNA was extracted from skeletal muscle samples using Reagent for ExtractRNA (BC032, an analog of TRIzol, Evrogen, Moscow, Russia) and subsequently treated with RNase-Free DNase (Magen Biotechnology Co., Guangzhou, China) in accordance with the manufacturer’s protocol to eliminate potential genomic DNA contamination. DNase was inactivated by adding EDTA to a final concentration of 0.017 M, followed by incubation at 70 °C for 10 min, and the samples were then purified with the Amplitech RNA-100 kit (Amplitech, Moscow, Russia). RNA integrity and concentration were assessed via spectrophotometry (EzDrop1000, Bluy-Ray Biotech, New Taipei City, Taiwan), agarose gel electrophoresis method and Agilent 2100 (Agilent Technologies, Santa Clara, CA, USA) analysis. All RNA samples had a high integrity, with an RNA Integrity Number (RIN) of no less than 7, ensuring the reliability of subsequent transcriptomic analysis. To address the problem of limited biological replicates, 18–23 individuals per group were included in the analysis, and their RNA was combined to create representative RNA pools for the library preparation.

The pooled RNA sampling strategy was used due to the significant logistical constraints of obtaining deep-water sculpin specimens from the abyssal zone of Lake Baikal. The primary objective was to identify constitutive transcriptional signatures underlying major ecological adaptations between pelagic and benthic lineages, rather than to assess individual variation. To ensure the robustness of our findings despite this approach, two different species were used within each ecological group. Moreover, we applied stringent statistical thresholds (*p* < 0.0001, logFC = 3) to identify high-confidence differentially expressed genes (see Section 2.4). In addition, the key transcriptional patterns identified were successfully validated using qPCR on unpooled individual biological replicates, confirming the reliability of the major interspecific differences reported (see Section 2.5).

### 2.3. Library Construction and Sequencing

The messenger RNA was separated from the total RNA using poly-T oligo-attached magnetic beads. Following mRNA fragmentation, first-strand cDNA synthesis was primed with random hexamers, and a second strand was synthesized using dTTP to generate a non-directional library. This library was then finalized through a series of preparative steps, including end repair, A-tailing, adapter ligation, size selection, amplification, and purification. The resulting libraries were quantified and assessed for size distribution using Qubit and real-time PCR, followed by equimolar pooling. High-throughput sequencing was performed by Novogene on an Illumina NovaSeq 6000 platform (Novogen Co., Beijing, China), using a NovaSeq 6000 Reagent Kit v.1.5 (Illumina, San Diego, CA, USA) to generate 150 bp paired-end reads. Base-call quality, expressed as Phred scores (Qphred) on the Illumina platform, was derived from the error probability (e) using the equation Qphred = −10log10(e). Finally, raw reads were filtered with Trimmomatic v.0.36 [31] to eliminate adapter sequences and low-quality sequences, yielding a refined set of clean reads for subsequent analysis.

### 2.4. De Novo Assembly and Differential Gene Expression Analysis

Due to the lack of a high-quality reference genome for these non-model species and their considerable phylogenetic divergence (approximately 2.5 million years), a de novo assembly approach was employed to obtain a comprehensive transcript catalog. Given that the studied species belong to distinct taxa, transcriptome assembly was performed separately for each species using Trinity v.2.13.2 software package [32]. The combined assembly was then processed using CD-HIT v.4.8.1 [33] to remove duplicate sequences and identify common genes. Sequences with ≥95% homology were clustered and reduced to unique representatives. To assign unique identifiers, numerical suffixes in ascending order were appended to all sequence names using a custom script (‘add_counters.js’, available at: https://github.com/tuyana-bot/add_counters, accessed on 26 November 2025). Subsequently, a gene-to-transcript map file (name.gene_trans_map), which correlates gene identifiers with their corresponding transcript (isoform) identifiers, was generated using the script ‘get_Trinity_gene_to_trans_map.pl’ (available at: https://github.com/tuyana-bot/get_Trinity_gene_to_trans_map, accessed on 26 November 2025). A transcriptome index for expression quantification was built using the command ‘salmon index -t name.fasta -i name_index’ (Salmon v.1.10.0) [34]. Transcript expression levels were estimated using the script ‘align_and_estimate_abundance.pl’. Next, the script ‘abundance_estimates_to_matrix.pl’ was used. This step converted the individual transcript expression estimates into a single matrix of counts, TPM, and FPKM values, suitable for subsequent differential expression analysis and data visualization.

Differential expression analysis was performed using the R package EdgeR v.4.0.3 [35]. EdgeR provides the statistical foundation for identifying differential expression by applying an overdispersed Poisson model, with an integrated empirical Bayes method employed to moderate the degree of overdispersion across genes [36]. The dispersion parameter was set to 0.2 to account for the anticipated degree of biological variability between samples. This value was selected because the studied individuals were wild-caught rather than reared in a controlled laboratory environment, and were therefore expected to exhibit greater variability in gene expression. Transcripts were initially considered differentially expressed (DE) using a significance threshold of *p*-value < 0.001 and an absolute logarithmic fold-change of 2 (logFC = 2), that is, at least a fourfold change in expression. The *p*-values were adjusted for multiple testing. This stringent parameter was used to reduce the likelihood of false positive results. In addition, to isolate the most prominent changes, the analysis was subsequently refined to focus on the highly differentially expressed genes (top-DEGs) through the implementation of more stringent filters (adjusted *p*-value < 0.0001, logFC = 3). A distance matrix was calculated using the Pearson correlation method to support the clustering of functionally similar genes [37], and the resulting relationships were visualized in a heatmap generated with the R package pheatmap v1.0.12. The identified differentially expressed genes (DEGs) were then annotated by homology search via the GenBank v.254 database using BLAST (https://www.ncbi.nlm.nih.gov/geo/query/blast.html, accessed on 26 November 2025), applying a stringent e-value threshold of *p*-value < 0.001 to ensure high-confidence functional assignments. Subsequent functional prediction and classification were conducted with the EggNOG mapper (orthologous groups of genes, http://eggnog5.embl.de, accessed on 26 November 2025) [38], followed by enrichment analysis performed using KOBAS software v.3.0 [39] in conjunction with Gene Ontology (GO) terms and Kyoto Encyclopedia of Genes and Genomes (KEGG) pathways. Terms and pathways with a corrected *p*-value < 0.05 were considered statistically significant, highlighting their potential biological importance.

### 2.5. Validation of Transcriptomic Data via qPCR

RNA extraction and subsequent preparation were performed as described in Section 2.2. cDNA was synthesized from the extracted RNA using random hexamer primers and the Reverta-L reagent kit (AmpliSens, Moscow, Russia), according to the manufacturer’s protocol.

The transcriptome data accuracy was validated on individual biological replicates (18–23 per species). Several highly expressed genes were selected for quantitative PCR (qPCR) analysis. The Recombination Activating Gene 1 (RAG1) served as the reference gene based on its documented stability in prior studies [12]. Primers were designed in Primer-BLAST (https://www.ncbi.nlm.nih.gov/tools/primer-blast/, accessed on 26 November 2025) and conformed to MIQE guidelines [40]. Primer specificity was confirmed by melting curve analysis, and through transcriptome alignment to verify efficiency. The primer sequences are listed in Table 3.

qPCR was conducted on a BIO RAD CFX96 Touch Real-Time PCR Detection System (BioRad, Hercules, CA, USA) using a reaction mixture composed of 0.25 mM dNTPs, 0.3 U 1× Encyclo-polymerase (Evrogen, Moscow, Russia), 1× Encyclo-buffer, 0.5× SYBR Green (Lumiprobe, Hunt Valley, MD, USA), 0.5–0.6 ng of cDNA, and 0.5 pmol of each primer. The reference and target genes were amplified using a touchdown PCR protocol, which involved progressively lowering the primer annealing temperature from 67 to 60 °C over the course of the first seven cycles. The thermal profile of each cycle began with DNA polymerase activation at 95 °C for 3 min, followed by 40 cycles of amplification (95 °C for 10 s, 60 °C for 15 s, and 72 °C for 15 s), with a melting curve analysis performed post-amplification to confirm product specificity.

Relative gene expression was determined via the ΔΔCq method using the BIO RAD system’s integrated software v.3.1. Statistical comparisons of expression levels were performed with the Kruskal–Wallis test in the Statistica 10 package, considering *p* < 0.05 as statistically significant.

## 3. Results

### 3.1. Transcriptome Sequencing and De Novo Assembly

**Figure A1:**
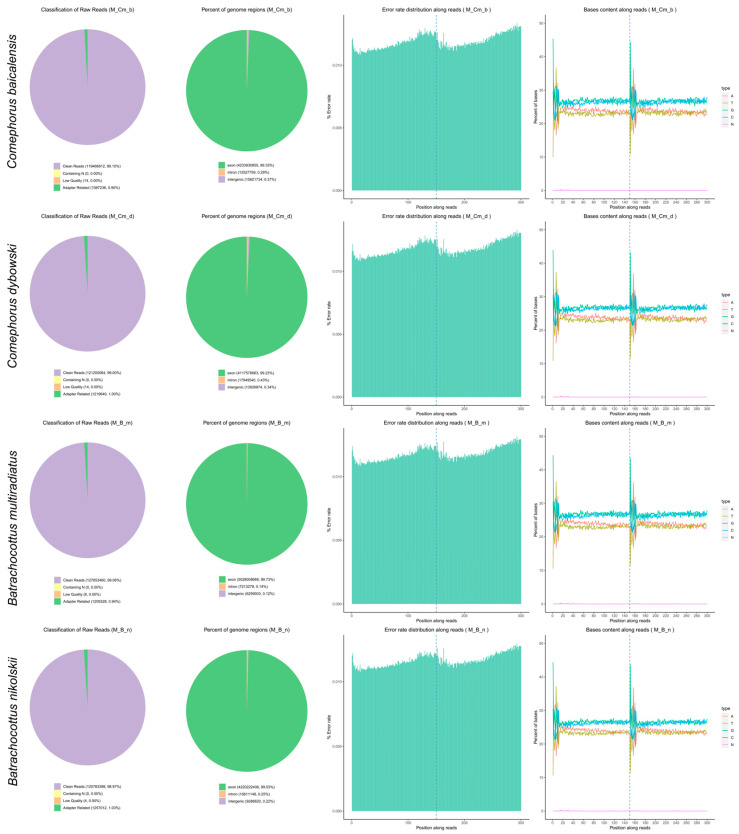
Sample sequencing overview, including data filtering, genomic region mapping of sequencing reads, sequencing data error rate distribution, and GC content distribution.

Sequencing quality metrics are summarized in Table 4, and the distribution of sequencing reads mapping to exons, introns, and intergenic regions across all samples is depicted in Figure A1. The non-stranded library preparation resulted in a balanced GC/AT nucleotide composition throughout sequencing (Table 4).

**Figure 2:**
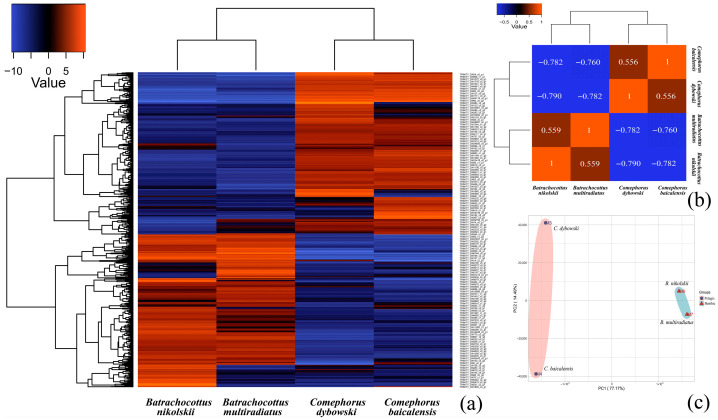
Analysis of differentially expressed genes (DEGs) in Baikal sculpins. (**a**)—a heatmap displays log_2_ fold-change (logFC = 2) values (*p* < 0.001). Genes are clustered by expression pattern similarity (Pearson correlation), with the color key indicating high (red) and low (blue) expression. Complete logCPM and logFC data are archived in Supplementary Materials S5 and S6. (**b**)—The Pearson correlation coefficient matrix shows division into pelagic and benthic groups. The closer the correlation coefficient is to 1, the higher similarity the expression patterns have. (**c**)—PCA analysis on the gene expression value (TPM) for pelagic and benthic groups. PC1 shows the primary adaptation to different ecological niches, while PC2 reflects species differences within each group.

All raw sequence data are available in the NCBI Gene Expression Omnibus (GEO) under accession number GSE308109 (https://www.ncbi.nlm.nih.gov/geo/query/acc.cgi?acc=GSE308109, accessed on 26 November 2025). A subsequent de novo transcriptome assembly generated 152,088 contigs with a total length of 121,871,112 bp and a maximum contig length of 11,944 bp, the details of which are available in Supplementary Materials S1 and S2; this assembly is also accessible within the same GEO repository. To identify transcriptional differences among Baikal sculpin species, a differential gene expression analysis was conducted. Applying a significance threshold of *p* < 0.001 (logFC = 2) when comparing all four species, a total of 793 differentially expressed genes (DEGs) were identified, as presented in Figure 2. Comprehensive lists of these DEGs, including functional annotations, are provided in Supplementary Materials S3, which catalogs all DEGs without a threshold, and S4 with a significance threshold set at *p*-value < 0.001 (logFC = 2).

**Figure 3:**
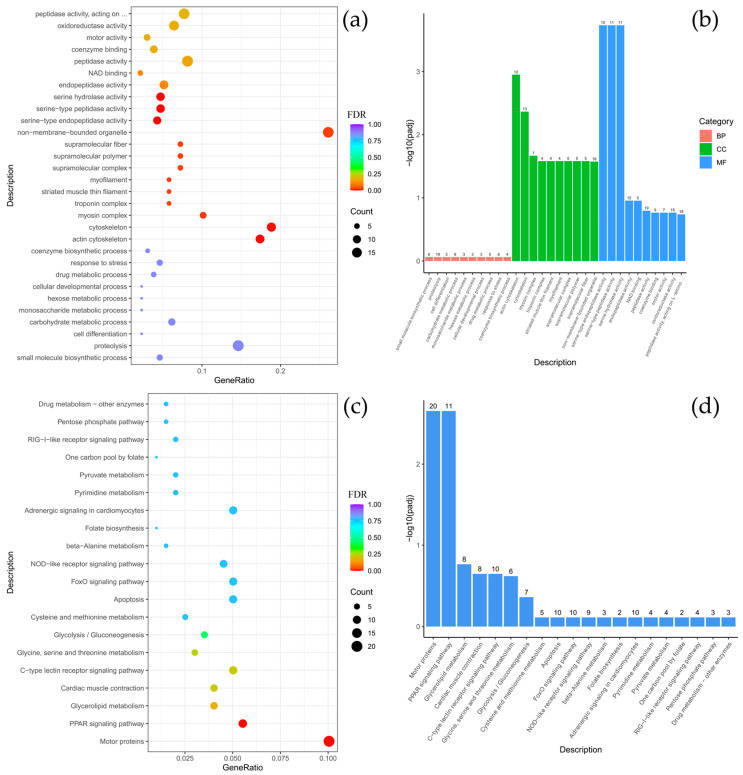
Comparison of *Comephorus baicalensis* and *Comephorus dybowski*: GO and KEGG enrichment analysis of DEGs. (**a**,**b**)—the most significant terms from the GO databases, including biological processes (BP), cellular components (CC), molecular function (MF); (**c**,**d**)—the top enriched terms for KEGG enrichment. The size of a point (**a**,**c**) and numbers above columns (**b**,**d**) represent the number of genes annotated to a specific term. Terms with FDR < 0.05 are considered significant.

**Figure 4:**
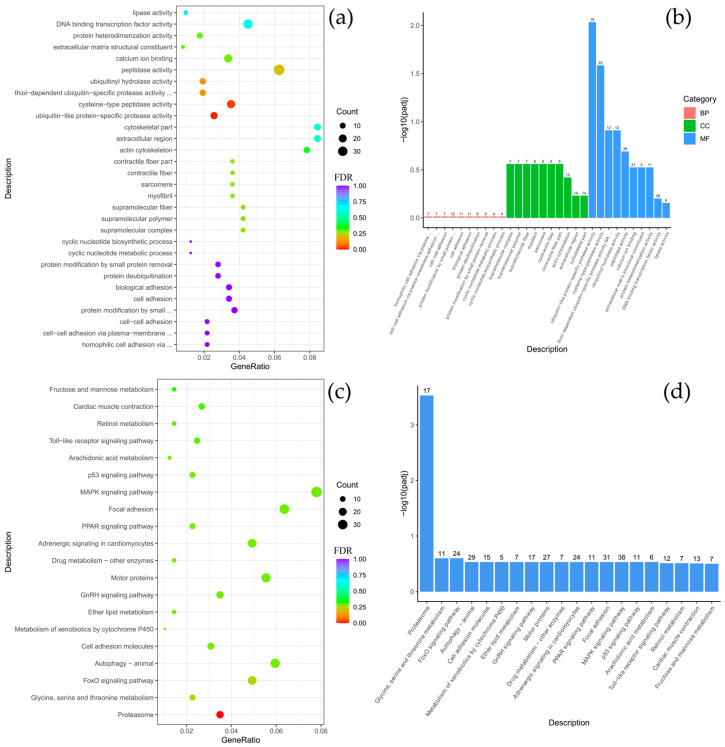
Comparison of *Batrachocottus multiradiatus* and *Batrachocottus nikolskii*: GO and KEGG enrichment analysis of DEGs. (**a**,**b**)—the most significant terms from the GO databases, including biological processes (BP), cellular components (CC), molecular function (MF); (**c**,**d**)—the top enriched terms for KEGG enrichment. The size of a point (**a**,**c**) and numbers above columns (**b**,**d**) represent the number of genes annotated to a specific term. Terms with FDR < 0.05 are considered significant.

**Figure A2:**
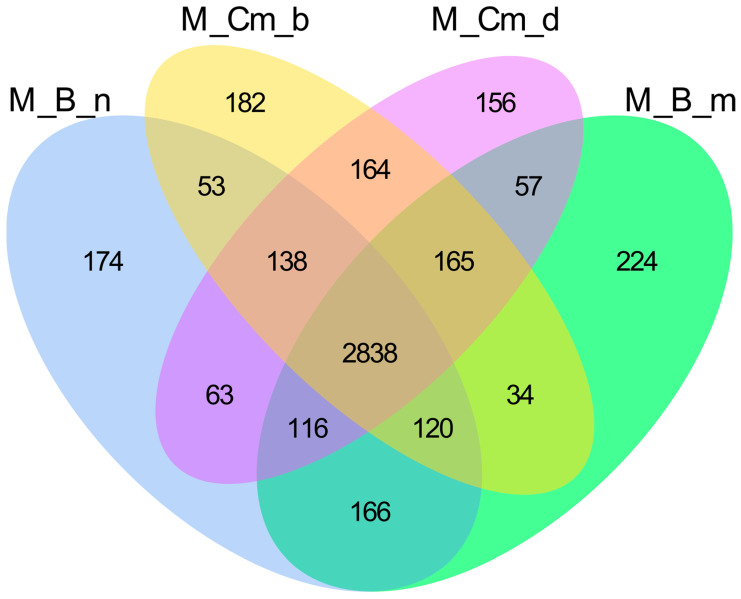
Venn diagram of gene co-expression across Baikal sculpin species. The diagram shows the number of uniquely expressed genes for each species, with overlapping areas indicating genes co-expressed in two or more groups. M_B_n—*Batrachocottus nikolskii*, M_Cm_b—*Comephorus baicalensis*, M_Cm_d—*Comephorus dybowski*, M_B_m—*Batrachocottus multiradiatus*.

**Figure A3:**
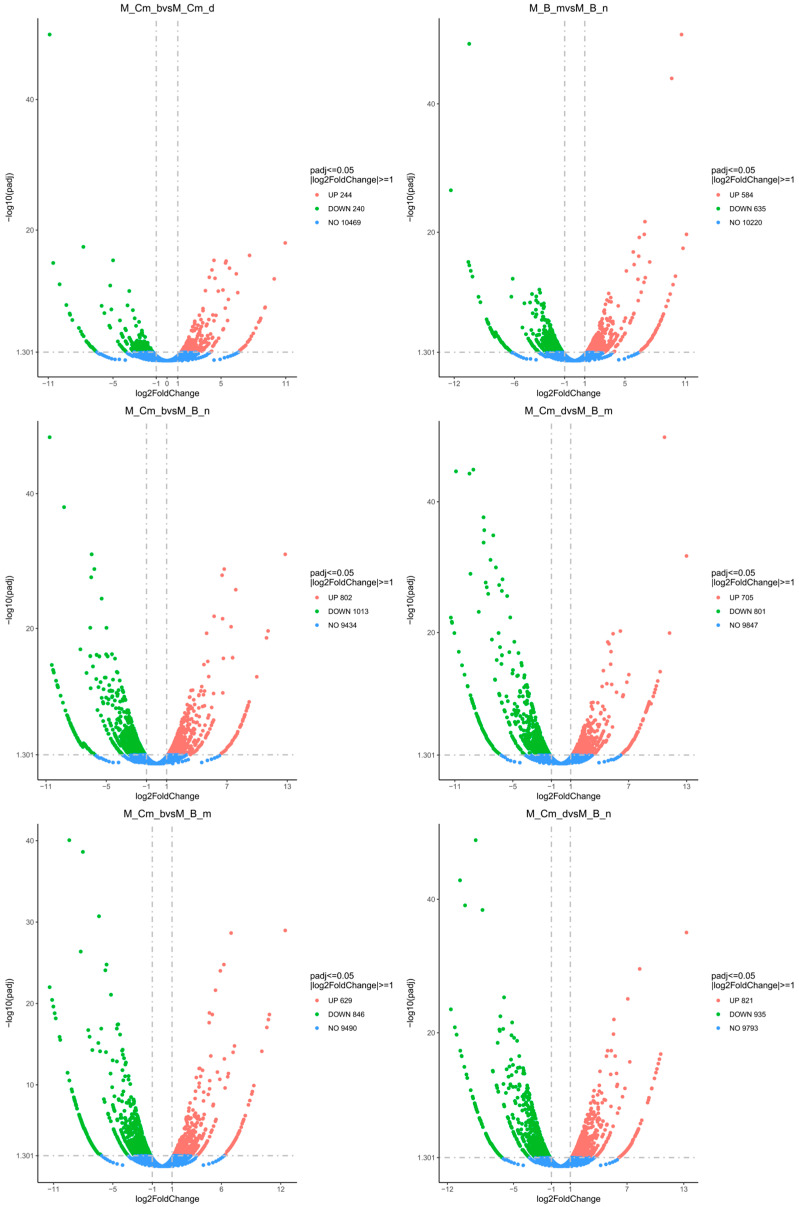
Volcano plots of differential gene expression in pairwise Baikal sculpin species comparisons. Red dots represent up-regulation genes and green dots represent down-regulation genes. The gray dashed line indicates the threshold line for differential gene screening criteria. M_B_n—*Batrachocottus nikolskii*, M_Cm_b—*Comephorus baicalensis*, M_Cm_d—*Comephorus dybowski*, M_B_m—*Batrachocottus multiradiatus*.

The statistical analysis showed that the differences between the ecological groups—pelagic (*C. baicalensis* and *C. dybowski*) versus benthic (*B. multiradiatus* and *B. nikolskii*)—were more pronounced than the differences between species within each group (Figure 2b,c). In addition, an analysis of the overall distribution of differentially expressed genes (DEGs) also showed the smallest number of differences in the pairwise comparison between the two species within the same genus (Figure A3). In contrast, comparisons between different genera revealed the highest number of DEGs, with a maximum of 821 up-regulated and 935 down-regulated DEGs identified between *C. dybowski* and *B. nikolskii* (Figure A2 and Figure A3). Nevertheless, species within the genus *Comephorus* exhibited greater divergence from each other than species within the genus *Batrachocottus* (Figure 2c), as confirmed by the comparison of significantly different terms and pathways in the GO and KEGG analyses (see Section 3.2: Figure 3 and Figure 4).

### 3.2. GO and KEGG Analysis of DEGs

To clarify the biological functions of the differentially expressed genes (DEGs) between *C. baicalensis* and *C. dybowski*, Gene Ontology (GO) and KEGG pathway enrichment analyses were performed. The GO analysis revealed a significant enrichment of DEGs in specific functional categories, particularly within the cellular component (CC) category for structural elements such as the cytoskeleton, myofilament, striated muscle thin filament, and associated complexes including myosin and troponin. There were classified under the broader terms of supramolecular polymers and fibers, as well as non-membrane-bounded organelles. In the molecular function (MF) category, there was notable enrichment for serine-based enzymatic activities, including serine-type endopeptidase and peptidase activity, as well as serine hydrolase activity (Figure 3a,b). The KEGG pathway analysis further indicated that the DEGs were significantly enriched in key signaling and functional pathways, with the highest gene count enrichment observed for motor proteins, followed by the peroxisome proliferator-activated receptor (PPAR) signaling pathway (Figure 3c,d).

Comparative analysis between *B. multiradiatus* and *B. nikolskii* revealed the least pronounced differential gene expression among the groups studied. GO enrichment analysis indicated that the differentially expressed genes (DEGs) were exclusively and highly enriched in the molecular function (MF) category, specifically for cysteine-type peptidase activity and ubiquitin-like protein-specific protease activity (Figure 4a,b). Supporting these findings, KEGG pathway analysis demonstrated significant enrichment for proteasome-related functions. Furthermore, the highest numbers of gene enrichments were identified in pathways critical for cellular signaling and homeostasis, including MAPK signaling, focal adhesion, adrenergic signaling in cardiomyocytes, motor proteins, autophagy, and the FoxO signaling pathway (Figure 4c,d).

**Figure 5:**
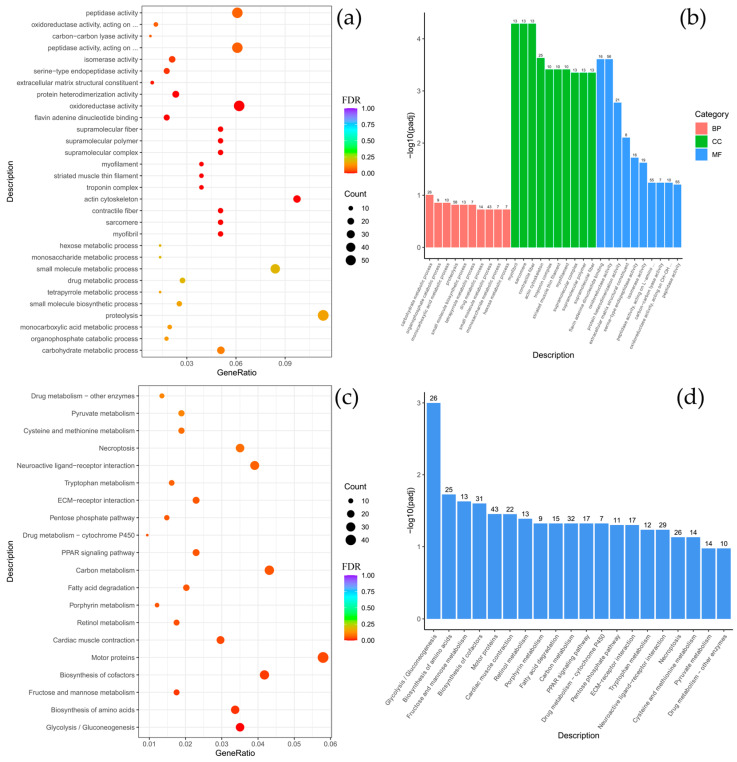
Comparison of *Comephorus dybowski* and *Batrachocottus nikolskii*: GO and KEGG enrichment analysis of DEGs. (**a**,**b**)—the most significant terms from the GO databases, including biological processes (BP), cellular components (CC), molecular function (MF); (**c**,**d**)—the top enriched terms for KEGG enrichment. The size of a point (**a**,**c**) and numbers above columns (**b**,**d**) represent the number of genes annotated to a specific term. Terms with FDR < 0.05 are considered significant.

**Figure A4:**
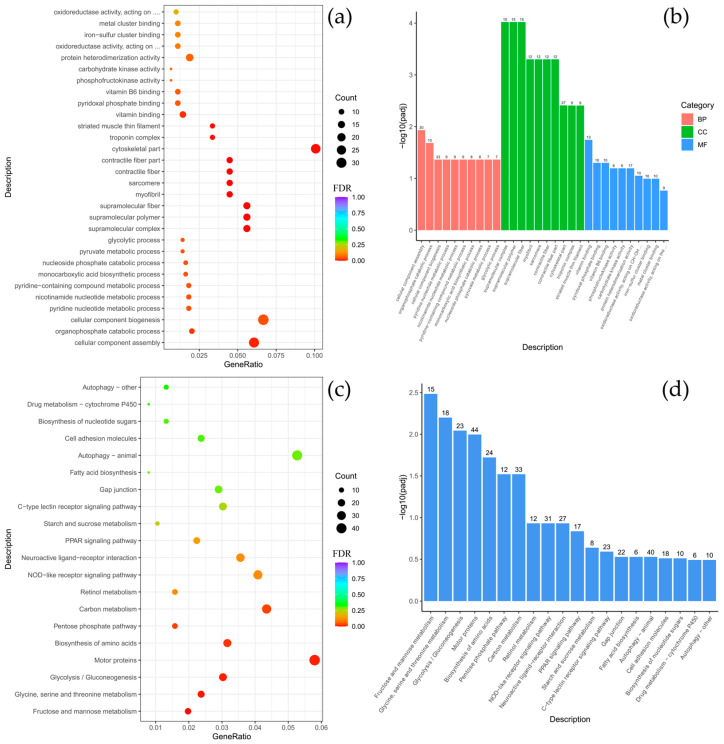
Comparison of *Comephorus baicalensis* and *Batrachocottus nikolskii*: GO and KEGG enrichment analysis of DEGs. (**a**,**b**)—the most significant terms from the GO databases, including biological processes (BP), cellular components (CC), molecular function (MF); (**c**,**d**)—the top enriched terms for KEGG enrichment. The size of a point (**a**,**c**) and numbers above columns (**b**,**d**) represent the number of genes annotated to a specific term. Terms with FDR < 0.05 are considered significant.

**Figure A5:**
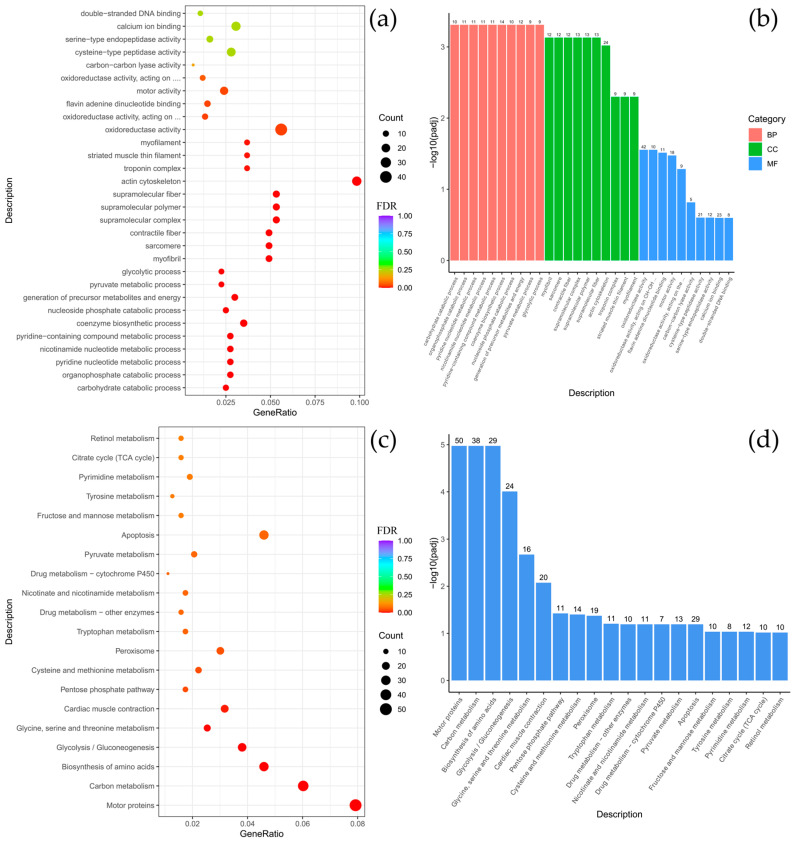
Comparison of *Comephorus dybowski* and *Batrachocottus multiradiatus*: GO and KEGG enrichment analysis of DEGs. (**a**,**b**)—the most significant terms from the GO databases, including biological processes (BP), cellular components (CC), molecular function (MF); (**c**,**d**)—the top enriched terms for KEGG enrichment. The size of a point (**a**,**c**) and numbers above columns (**b**,**d**) represent the number of genes annotated to a specific term. Terms with FDR < 0.05 are considered significant.

**Figure A6:**
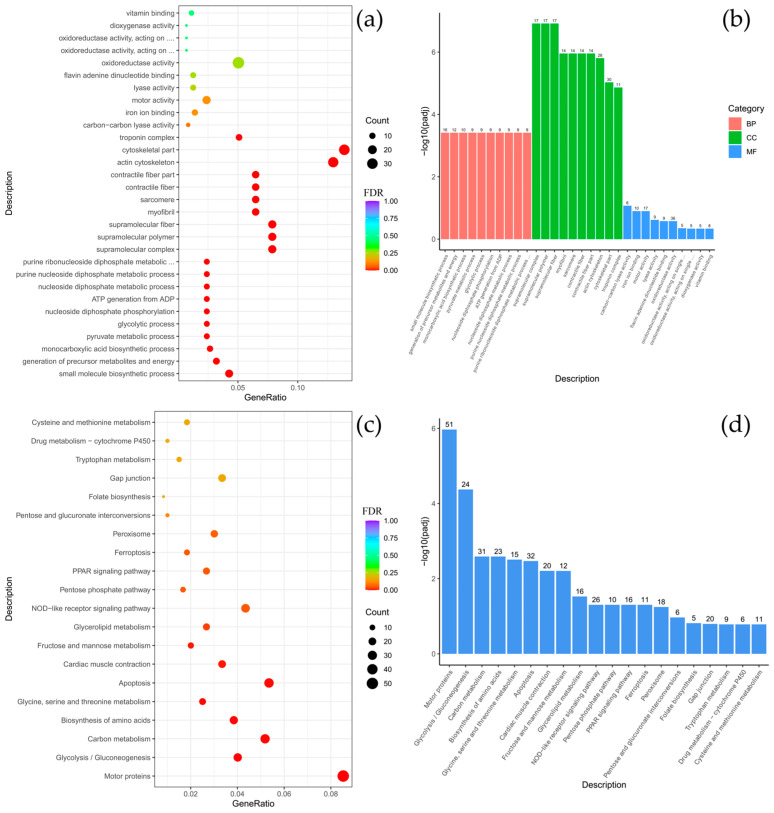
Comparison of *Comephorus baicalensis* and *Batrachocottus multiradiatus*: GO and KEGG enrichment analysis of DEGs. (**a**,**b**)—the most significant terms from the GO databases, including biological processes (BP), cellular components (CC), molecular function (MF); (**c**,**d**)—the top enriched terms for KEGG enrichment. The size of a point (**a**,**c**) and numbers above columns (**b**,**d**) represent the number of genes annotated to a specific term. Terms with FDR < 0.05 are considered significant.

As expected, comparative analysis between different fish genera inhabiting distinct ecological niches revealed greater divergence in functional and pathway annotations than comparisons between species of the same genus living in similar conditions. Specifically, the comparison between species of the genera *Batrachocottus* and *Comephorus* identified DEGs significantly enriched in the following categories: within the biological process (BP) category, enrichment was predominantly observed for glycolytic and pyruvate metabolic processes. Concurrently, in the cellular components (CC) category, significant enrichment was observed for genes associated with the troponin complex, contractile fibers, the sarcomere, myofibrils, and various supramolecular structures, including fibers, polymers, and complexes (Figure 5, Figure A4, Figure A5 and Figure A6).

Further interspecific comparisons highlighted species-specific functional specializations. The contrast between *C. dybowski* and benthic *Batrachocottus* species demonstrated that the largest number of DEGs was associated with oxidoreductase activity (Figure 4a,b and Figure A5a,b). In contrast, *C. baicalensis* displayed a different pattern when compared with the same benthic *Batrachocottus* species, with significant enrichment of genes related to the cytoskeleton, specifically those annotated to cytoskeletal part processes (Figure A4a,b and Figure A6a,b).

According to the KEGG analysis, DEGs were enriched in pathways such as carbon metabolism, pentose phosphate pathway, biosynthesis of amino acids, motor proteins, glycolysis/gluconeogenesis, and fructose and mannose metabolism when comparing species of the genera *Batrachocottus* and *Comephorus*. The highest number of enrichments was observed in the pathways of carbon metabolism, biosynthesis of amino acids, motor proteins, and glycolysis/gluconeogenesis (Figure 5c,d, Figure A4c,d, Figure A5c,d and Figure A6c,d).

### 3.3. Analysis of Top-DEGs: Key Metabolic and Structural Genes

**Figure 6:**
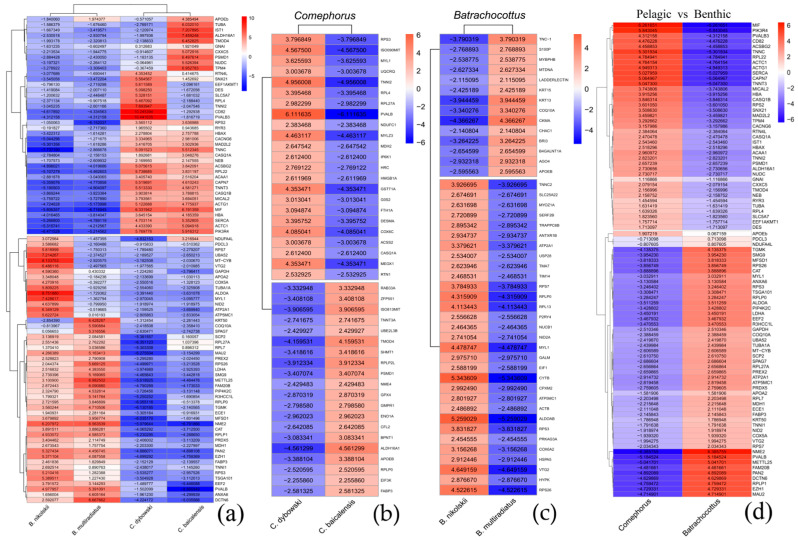
The top-DEGs in Baikal sculpins. The heatmap shows the log_2_ fold-change (logFC = 3, *p* < 0.0001). (**a**–**c**)—Transcripts are clustered according to similar expression patterns in the groups (Pearson correlation method); (**d**)—Hierarchical clustering of transcripts based on Euclidean distance. Red indicates genes with high expression levels (up-regulated DEGs), and blue indicates genes with low expression levels (down-regulated DEGs). (**a**)—Comparison of all four species; (**b**)—Comparison of *Comephorus* species; (**c**)—Comparison of *Batrachocottus* species; (**d**)—Comparison of two different genera, the benthic-abyssal *Batrachocottus* and the pelagic *Comephorus*.

To delineate the most robust transcriptional signatures in Baikal sculpins, the significance threshold was stringently set at *p*-value < 0.0001 (logFC = 3) to identify the top differentially expressed genes (top-DEGs). Their expression profiles clearly separate the pelagic and benthic genera (Figure 6).

Transcriptomic analysis of muscle tissue revealed distinct expression profiles between the two genera of deep-water sculpin fishes, *Comephorus* and *Batrachocottus* (Figure 6). In general, the pelagic *Comephorus* species (*C. dybowski* and *C. baicalensis*) exhibited pronounced upregulation of genes associated with the sarcomeric complex and oxygen transport. Both species showed high expression levels of actin cytoplasmic (*ACTG1*), actin alpha cardiac muscle (*ACTC1*), F-actin-monooxygenase (*MICAL2*), troponin C (*TNNC*) and troponin T (*TNNT3*), indicating specializations in muscle contraction dynamics. Troponin I fast skeletal muscle (*TNNI2*) was upregulated in *C. dybowski*, while tropomyosin 4 (*TPM4*) and tropomodulin (*TMOD4*) showed high expression in *C. baicalensis*. Furthermore, elevated expression of hemoglobin subunit alpha (*HBAX*) was observed. The gene encoding reticulon 4 (*RTN4L*) was also significantly upregulated in *C. dybowski*, suggesting potential neural or endoplasmic reticulum specializations.

In contrast, the benthic species of the *Batrachocottus* genus displayed a markedly different transcriptomic signature, characterized by enhanced expression of genes involved in mitochondrial energy metabolism and lipid utilization. The deep-water benthic *B. nikolskii* showed dramatic upregulation of cytochrome b (*MT-CYB*) and cytochrome c oxidase (*COX5A*), key components of the mitochondrial electron transport chain. This species also exhibited elevated levels of fatty acid-binding protein 3 (*FABP3*) and apolipoprotein A-II (*APOA2*), which facilitate intracellular fatty acid transport. The eurybenthic *B. multiradiatus* showed a similar trend in lipid metabolism, with high expression of FABP3 and phosphatidylinositol-5-phosphate 4-kinase type 2 gamma (*PIP4K2C*), but was uniquely characterized by strong upregulation of keratin 50 (*KRT50*).

Several genes demonstrated reciprocal expression patterns that further highlight divergent ecological adaptations. For example, troponin T (*TNNT3*), hemoglobin subunit alpha (*HBAX*), guanine nucleotide-binding protein (*GNAI*), and actin cytoplasmic (*ACTG1*) were highly expressed in *Comephorus*, but strongly downregulated in *Batrachocottus*. Conversely, genes such as fatty acid-binding protein 3 (*FABP3*), endothelin converting enzyme (*ECE1*) and phosphatidylinositol-5-phosphate 4-kinase type 2 gamma (PIP4K2C) were upregulated in the benthic species but downregulated in the pelagic ones. The glycolytic enzymes, malate dehydrogenase 1 (*MDH1*) and L-lactate dehydrogenase A (*LDHA*), were also downregulated in both *Comephorus* species but upregulated in *Batrachocottus*, suggesting differences in metabolic potential or a possible switch to other mechanisms of energy production in *Comephorus* species, as confirmed by activation of the PPAR signaling pathway in these species (Figure 3c,d). Similarly, nidogen 2 (*NID2*) and phosducin-like 3 (*PDCL3*) were upregulated in *B. nikolskii*, indicating potential specializations in extracellular matrix organization and G-protein signaling, respectively, which are less prominent in the pelagic genus.

In summary, the transcriptomic profiles clearly differentiate the pelagic *Comephorus* genus, which emphasizes muscle contraction and oxygen transport genes, from the benthic *Batrachocottus* genus, which shows increased expression of genes involved in cellular resilience and lipid metabolism.

### 3.4. Validation of Transcriptomic Data via qPCR

**Figure 7:**
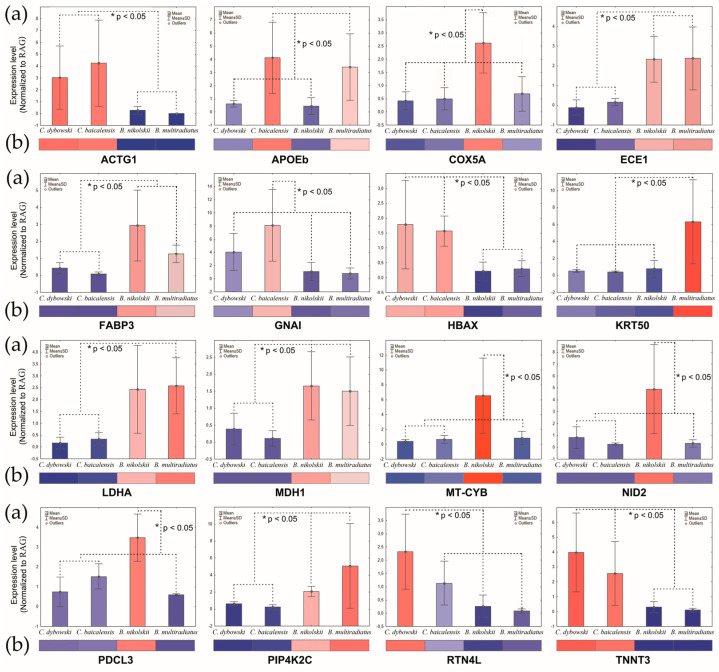
Comparison of the expression levels of DEGs detected by qPCR (**a**) and the data of gene-corresponding patterns from RNA-seq (**b**) in Baikal sculpins. Significant statistical differences between the species are indicated by an asterisk: * *p* < 0.05 (Kruskal–Wallis test).

The expression of key genes was validated using quantitative PCR (qPCR). The qPCR results (Figure 7a) exhibited a strong concordance with the RNA-Seq data (Figure 6 and Figure 7b), thereby confirming the reliability of the transcriptomic findings.

## 4. Discussion

### 4.1. The Pelagic Signature: Rewiring Musculoskeletal and Metabolic Systems for a Life in the Water Column

The pelagic *Comephorus* genus, which undergoes diel vertical migration through the oxygenated bathyal zone, showed significant upregulation of genes encoding structural and contractile proteins (Figure 6 and Figure 7). These included actin cytoplasmic (*ACTG1*, logFC = 4.78 in *C. baicalensis* and logFC = 5.12 in *C. dybowski*), actin alpha cardiac muscle (*ACTC1*, logFC = 5.09 and logFC = 4.43, respectively), F-actin-monooxygenase (*MICAL2*, logFC = 3.69 and logFC = 3.79, respectively), troponin C (*TNNC*, logFC = 5.51 and logFC = 5.09, respectively) and troponin T (*TNNT3*, logFC = 4.58 and logFC = 5.51, respectively) in both species. We also observed upregulation of troponin I fast skeletal muscle (*TNNI2*, logFC = 7.69) in *C. dybowski*, and tropomyosin 4 (*TPM4*, logFC = 6.95) and tropomodulin (*TMOD4*, logFC = 6.45) in *C. baicalensis*. This pattern indicates specialized skeletal muscle physiology. This conclusion is further supported by the increased expression of hemoglobin alpha (*HBAX*, logFC = 2.76 and logFC = 2.28, respectively) in both species of *Comephorus*, which aligns with previous physiological and biochemical data on hemoglobin fractions [13]. This adaptation likely enhances oxygen saturation in the cold pelagic environment by improving blood oxygen affinity. This mechanism could compensate for the reduced gill surface area in *Comephorus* [41], which is considered an energy-saving adaptation [42].

The upregulation of key structural and contractile elements indicates a musculoskeletal system specialized for continuous locomotion and maneuverability in a three-dimensional environment. This represents a major shift from the benthic “sit-and-wait” strategy and corresponds with the highly derived morphology of *Comephorus* species. This is evidenced by their elongated, laterally flattened body; a head lacking armament; long and broad, fan-shaped pectoral fins; flexible skull bones; and a lightweight skeleton. Their diet also reflects this pelagic lifestyle. Adults primarily consume the pelagic amphipod *Macrohectopus branickii* (Dybowsky, 1874), which they hunt by performing vertical migrations to follow their prey, as well as juvenile fish of their own genus. The juveniles feed on the copepod *Epischura baikalensis* (Sars, 1900) [3,6,43]. This molecular profile is further reinforced by the upregulation of calcium-handling genes such as calsequestrin-1a (*CASQ1A*, logFC = 3.05 in *C. baicalensis* and logFC = 1.89 in *C. dybowski*), calsequestrin-1b (*CASQ1B*, logFC = 3.79 and logFC = 3.90, respectively) and sarcoplasmic/endoplasmic reticulum calcium ATPase 1 (*SERCA/ATP2A*, logFC = 5.35 and logFC = 4.70, respectively), which are crucial for the efficient cycling required for sustained muscle contraction (Figure 6). The enrichment of the “motor proteins” KEGG pathway supports the idea that efficient muscle function under high hydrostatic pressure is a key adaptation for pelagic life. This molecular signature of enhanced contractile machinery and calcium cycling is also characteristic of active fish species with a swim bladder and has been documented in other comparative studies of fish with varying activity levels [44].

We also found distinct transcriptional profiles between *C. dybowski* and *C. baicalensis*, indicating species-specific specializations. The most notable divergence between the two species is in their muscle contractile and calcium-handling machinery. The marked up-regulation of parvalbumin 3 (*PVALB3*, logFC = 10.44) and fast skeletal troponin I (*TNNI2*) in *C. dybowski* suggests an adaptation for rapid calcium cycling and potentially faster contraction kinetics (Figure 6). Parvalbumin acts as a soluble calcium buffer, accelerating muscle relaxation, which is crucial for sustained swimming [45]. In contrast, the up-regulation of tropomodulin-4 (*TMOD4*) in *C. baicalensis*, a regulator of actin filament length and stability, suggests a different strategy, possibly favoring structural stability over speed. This dichotomy indicates that even within the same genus and habitat, divergent evolutionary paths can be taken for muscle performance. The down-regulation of several ribosomal protein genes in *C. dybowski*, along with the up-regulation of contractile proteins, may reflect a shift in metabolic investment from protein synthesis to the specialized function of the muscle apparatus.

Another finding is the significant up-regulation of apolipoprotein Eb (*APOEb*, logFC = 4.39) in *C. baicalensis* (Figure 6 and Figure 7). This is consistent with previously obtained data on the lipid composition of species from this genus [46]. In particular, among the secondary pelagic fishes of Lake Baikal, only *C. baicalensis* is characterized by a high lipid content, constituting 38.9% of its wet body mass, whereas this value is only 4.7% in *C. dybowski* [47]. Our data indicate that this high lipid content in *C. baicalensis* is likely supported by the upregulation of mitochondrial genes involved in electron transport and energy metabolism, such as NADH:ubiquinone oxidoreductase subunits (e.g., *NDUFA4L*, logFC = 3.22) and ATP synthase subunits (*ATP5F1B*, logFC = 4.93) (Figure 6). This suggests a multi-level genomic rewiring of energy balance to support their lifestyle across a wide range of depths and pressures. This may be explained by the fact that sculpins lacking a swim bladder have evolved various adaptations for inhabiting the water column, including increased lipid deposition to reduce overall body density [46]. In the marine context, deep-sea fishes often exhibit substantial lipid deposition as a buoyancy adaptation, reducing their metabolic cost of maintaining position in the water column, a well-documented strategy in lieu of a swim bladder [48].

Although *C. dybowski* has a lower total body lipid content than *C. baicalensis*, the transcriptomic profiles suggest this difference may result from a divergence in lipid metabolism dynamics. This is supported by the concurrent up-regulation in *C. dybowski* of key enzymes involved in mitochondrial fatty acid β-oxidation, such as long-chain-fatty-acid-CoA ligase (*ACSBG2*, logFC = 5.08), which activates fatty acids for degradation, and 3-ketoacyl-CoA thiolase (*ACAA1*, logFC = 3.41), a terminal enzyme in the β-oxidation spiral (Figure 6). Moreover, *ACAA1* may elevate the proportion of polyunsaturated fatty acids (PUFAs) within membrane phospholipids [49,50,51] and potentially preserve essential membrane fluidity and functionality even at near-freezing temperatures [52,53]. The data suggest that *C. dybowski* maintains a state of high lipid flux and turnover, necessitating efficient transport and mobilization, rather than long-term storage, representing a synergistic solution to high hydrostatic pressure stressors in the water column. The high lipid content in *C. baicalensis* likely represents a more static energy reserve. This is confirmed by the high activity of the peroxisome proliferator-activated receptor (PPAR) signaling pathway (Figure 3), which plays a crucial role in systemic cell metabolism, regulating lipid expenditure, energy homeostasis, and immune response inhibition [54,55,56].

Finally, we observed adaptations for managing cellular stress in the deep pelagic zone. The up-regulation of glutathione S-transferase theta-1a (*GSTT1A*, logFC = 4.35) in *C. baicalensis* (Figure 6b) implies a reinforced system for detoxification and oxidative stress management. The significant upregulation of the pleiotropic macrophage migration inhibitory factor in both *Comephorus* species (*MIF*, logFC = 6.19, logFC = 6.33) may represent another layer of adaptation, potentially regulating glucose metabolism and cellular stress response [57]. This is a common challenge for deep-water organisms, which must cope with increased oxidative stress potentially induced by hydrostatic pressure [53,58]. The parallel up-regulation of cytochrome c oxidase (*COX6C*, logFC = 4.09), the terminal complex of eukaryotic oxidative phosphorylation in mitochondria, in *C. baicalensis* (Figure 6b) further suggests species-specific fine-tuning of aerobic respiration. Such modifications in the electron transport chain components are known to be sensitive to hydrostatic pressure and could represent an adaptation to maintain efficient ATP production under deep-water conditions [52].

The data reveal a compelling set of candidate genes for deep-water pelagic adaptation in freshwater sculpins. The obtained patterns observed, involving key structural and contractile elements, calcium-mediated muscle contractility, lipid transport, stress response, and mitochondrial efficiency, closely mirror the functional categories identified in studies of marine deep-sea species. This suggests a degree of convergent molecular evolution across distinct deep-water environments, both marine and freshwater.

### 4.2. The Benthic Blueprint: Metabolic Flexibility and Cellular Resilience in the Abyssal Zone

The benthic *Batrachocottus* species exhibited profound adaptations in metabolism and cellular homeostasis. Significant enrichment of pathways such as carbon metabolism, glycolysis/gluconeogenesis, including aldolase A (*ALDOA*) and glyceraldehyde-3-phosphate dehydrogenase (*GAPDH*), as well as the pentose phosphate pathway was especially pronounced in the deep-benthic specialist *B. nikolskii* (logFC = 8.75 and logFC = 4.59, respectively) (Figure 4, Figure 5 and Figure 6). This profile should not be interpreted as a high metabolic rate, but rather as a signature of metabolic flexibility. This species, restricted to deep soft bottoms with abundant detritus [3,43], appears to have developed this metabolic plasticity to efficiently capitalize on nutrient inputs. It has also been suggested that *B. nikolskii* can employ a hunting strategy involving brief bursts of speed. Although the primary diet of adult *B. nikolskii* consists of benthic gammarids and fish, juveniles of pelagic *Comephorus* species are a common prey item, occurring in up to 20% of samples [43]. The mechanism by which this sedentary species, as observed with the help of “Pisces” and “Mir” deep-water submersibles, captures such pelagic prey remains unclear and requires further investigation [15,43]. The ability to rapidly utilize available carbon sources through glycolysis and gluconeogenesis is likely crucial for feeding behavior and survival in environments where energy conservation is essential. Genes such as aldolase a fructose-bisphosphate b (*ALDOAB*, logFC = 8.75), which is highly upregulated in *B. nikolskii*, also indicate adaptations in glycolytic flux. The upregulation of cytochrome b (*MT-CYB*, logFC = 8.13) and cytochrome c oxidase (*COX5A*, logFC = 4.27) further highlights optimization of oxidative phosphorylation under high hydrostatic pressure, maintaining energy homeostasis in an energy-limited environment (Figure 6 and Figure 7). This is complemented by significant upregulation of genes central to muscle contraction and calcium handling, including myosin light chain 1 (*MYL1*, logFC = 7.43), tubulin alpha (*TUBA1A*, logFC = 5.81) and sarcoplasmic/endoplasmic reticulum calcium ATPase 1 (*ATP2A1*, logFC = 6.35) (Figure 6). SERCA1, encoded by *ATP2A1*, is critical for the reuptake of calcium into the sarcoplasmic reticulum, enabling rapid relaxation cycles in skeletal muscle [59], which is necessary for benthic predators.

The comparison between the deep-water benthic *B. nikolskii* and the eurybathic *B. multiradiatus* reveals a set of candidate genes that may underlie their distinct ecological specializations. The transcriptomic profile of *B. multiradiatus* suggests adaptations for a more active lifestyle across a broader depth range. The observed upregulation of creatine kinase (*CKMA*, logFC = 4.37) in *B. multiradiatus* aligns with its more active lifestyle (Figure 6c). Creatine kinase plays a pivotal role in cellular energy homeostasis by facilitating the rapid regeneration of ATP from phosphocreatine [60]. In a eurybathic predator, increased *CKMA* expression may represent an adaptation for maintaining energy efficiency during activities such as prey capture, ensuring rapid ATP availability for muscle contraction without requiring a high baseline metabolic rate. This finding is consistent with metabolic adaptations observed in other deep-water organisms [23]. The upregulation of the mitochondrial coenzyme Q-binding protein COQ10 homolog A (*COQ10A*, logFC = 5.59), which is involved in the electron transport chain, further supports a metabolic phenotype geared toward robust oxidative phosphorylation to meet higher energy demands (Figure 6a,c). The stress-responsive gene *BRI3* (logFC = 3.26), which was also upregulated in this species (Figure 6c), is associated with mechanisms that optimize mitochondrial function and mitigate cellular stress [61]. In addition, the eurybenthic *B. multiradiatus* showed high expression of phosphatidylinositol-5-phosphate 4-kinase type 2 gamma, which potentially may safeguard against pressure-induced cytoskeletal collapse and membrane damage [50], (*PIP4K2C*, logFC = 4.53), as well as in the closely related *B. nikolskii* (*PIP4K2C*, logFC = 2.32), but was uniquely characterized by strong upregulation of keratin 50 (*KRT50*, logFC = 6.43) (Figure 6a,c and Figure 7). Thus, the upregulation of the identified gene expression profile in *B. multiradiatus* indicates a different, eurybathic adaptation favoring activity.

The transcriptional patterns obtained in both *Batrachocottus* species are remarkably consistent with the distinct muscle biochemical phenotypes in benthic-abyssal Baikal sculpins described by Radnaeva et al. [62] and are supported by research in other systems [60]. In particular, changes in the activity of the following key enzymes may indicate a shift toward MUFA and homeoviscous adaptation in *B. nikolskii* and *B. multiradiatus*: sterol carrier protein (*SCP2*, logFC = 3.14 and logFC = 2.08, respectively), fatty acid-binding protein (*FABP3*, logFC = 2.46 and logFC = 1.83, respectively) in both species (Figure 6 and Figure 7), apolipoprotein A-II (*APOA2*, logFC = 3.35) in *B. nikolskii* and apolipoprotein Eb (*APOEb*, logFC = 1.97) in *B. multiradiatus*. Thus, the observed metabolic plasticity in deep-water organisms may be compensated by homeoviscous adaptation of cell membranes [63,64,65].

This adaptation to pressure is complemented by a strong enrichment of proteasome-related functions and ubiquitination pathways, such as E3 ubiquitin-protein ligases. Notably, there is pronounced upregulation of ribosomal protein genes in both *Batrachocottus* species, including 40S ribosomal protein S26 (*RPS26*, logFC = 2.04 and logFC = 5.67, respectively), 40S ribosomal protein S3 (*RPS3*, logFC = 5.21 and logFC = 1.28, respectively), 60S acidic ribosomal protein P0 (*RPLP0*, logFC = 2.72 and logFC = 3.85, respectively), as well as ubiquitin ribosomal protein eL40 (*UBA52*, logFC = 7.21) in *B. nikolskii*. These findings align with earlier ultrastructural observations showing an extensive organellar network and a high abundance of ribosomes in the cells of *B. nikolskii* compared to pelagic sculpin species [66]. These findings indicate a heightened capacity for protein synthesis [67] and the presence of DNA repair mechanisms, underscoring the critical importance of maintaining proteostasis and genomic integrity under the protein-denaturing conditions of high pressure in the abyssal zone [68,69,70,71].

Additionally, the transcriptomic data revealed several key antioxidant-related genes that were significantly upregulated in both *B. nikolskii* and *B. multiradiatus*. Notably, genes encoding enzymes such as catalase (*CAT*, logFC = 3.89 and logFC = 3.88, respectively) and peroxiredoxin-5 (*PRDX5*, logFC = 3.40 and logFC = 2.11, respectively) showed increased expression, indicating enhanced antioxidant defense mechanisms in these species (Figure 6). The maintenance of mitochondrial function under high hydrostatic pressure, whether optimized for efficiency in *B. nikolskii* or for higher output in *B. multiradiatus* combined with the protein-denaturing effects of this environment, can promote the generation of reactive oxygen species (ROS). As a result, the simultaneous enhancement of the antioxidant system serves as a critical compensatory adaptation to mitigate oxidative stress and protect cellular integrity. This finding is consistent with the increased antioxidants capacity and broader physiological challenges observed in other deep-water organisms, such as the Mariana Trench snailfish *Pseudoliparis swirei* (Gerringer and Linley, 2017), which is adapted to the hadal zone at depths greater than 7000 m [58,72].

In summary, the analysis reveals distinct molecular strategies for deep-water adaptation in two closely related sculpins. The benthic-abyssal specialist *B. nikolskii* displays a transcriptional profile focused on energy conservation and metabolic flexibility, optimizing pathways for opportunistic nutrient utilization (e.g., glycolysis and gluconeogenesis), efficient oxidative phosphorylation, and rapid muscle contraction. In contrast, the eurybathic *B. multiradiatus* demonstrates adaptations for a more active lifestyle, characterized by molecular machinery for rapid energy turnover and robust oxidative metabolism. The distinct regulatory state of the FoxO signaling pathway (Figure 4) between these species likely orchestrates their divergent metabolic and stress-response strategies, reflecting their different ecological constraints. Despite these differences, both species share compensatory adaptations to universal deep-water challenges, including enhanced antioxidant defenses to mitigate oxidative stress and increased protein synthesis and ubiquitination mechanisms to maintain proteostasis under high pressure. These transcriptional differences provide a foundation for understanding the molecular mechanisms that underpin niche partitioning in closely related species inhabiting distinct environments.

### 4.3. Parallel Paths in Adaptation: Baikal Sculpins in a Global Context

The evolutionary trajectory of deep-water teleosts, historically divided into ancient forms (e.g., Gadiformes) and secondary colonists (e.g., Ophidiidae and Liparidae) living at depths over 6000 m [73], provides perspective for interpreting the transcriptional adaptations of deep-water sculpins of Lake Baikal. Our findings show that Baikal freshwater sculpins, as secondary deep-water colonists, have converged on molecular solutions remarkably similar to those of their marine counterparts [22,23,24,26,50,51,52,53,58,73,74,75,76].

The specialization of the pelagic *Comephorus* species for locomotion is highlighted by significant enrichment of genes encoding cytoskeletal, myofilament, and troponin complex proteins. This emphasis on the contractile apparatus aligns with research on the evolutionary adaptation of functional genes to high pressure, which identified unique amino acid substitutions in α-skeletal actin (*ACT*) and myosin heavy chain (*MyHC*) proteins in deep-water fish [77,78,79]. For example, studies on the hadal snailfish *P. swirei* also revealed adaptations in protein turnover and muscle contraction genes with specific mutations in myosin heavy chains related to low temperature and high pressure [49,58]. Moreover, this focus coincided with analyses of 36 bathypelagic and abyssopelagic fish species that identified genes involved in responding to mechanical forces, such as *PIK3CA* and *VCL*, as targets of positive selection in deep-water fishes [50]. This convergence is further supported by studies on the deep-sea fish *Aldrovandia affinis* (Günther, 1877), where positively selected genes were implicated in microtubule regulation, a system known to be highly sensitive to hydrostatic pressure [23]. Interestingly, the upregulation of key contractile and calcium-cycling genes (e.g., *PVALB3*, *TNNI2*) in *Comephorus* is functionally analogous to the transcriptional responses observed in exercised the rainbow trout *Oncorhynchus mykiss* (Walbaum, 1792), where sustained swimming activity upregulates critical genes for muscle development and function, including myosin heavy chain (*MyHC*) and myosin light chain (*MyLC*) [20]. Nevertheless, the similar specialization of the contractile apparatus in *Comephorus* apparently supports not high-speed swimming, but rather a metabolic and muscular machinery essential for sustained fin flapping and position holding in the water column.

For benthic specialists, particularly *B. nikolskii*, the focus shifts toward metabolic flexibility and cellular integrity. The enrichment of glycolytic and proteasome pathways indicates an adaptation for efficient energy use and rigorous protein turnover in a stable, low-temperature environment. Key muscular enzymes such as lactate dehydrogenase (*LDH*) and malate dehydrogenase (*MDH*), present in the transcriptomic signature of both *Batrachocottus* species, show adaptively altered expression levels in other deep-water species, such as the black scabbardfish *Aphanopus carbo* (Lowe, 1839) [22,80,81,82].

The strong emphasis on proteostasis, including ubiquitination pathways, is also a recognized evolutionary response to protein-denaturing conditions. This is evidenced in the deep-sea mussels *Bathymodiolus platifrons* (Hashimoto and Okutani, 1994) and *Gigantidas platifrons* (Hashimoto and Okutani, 1994), which exhibits positive selection in genes linked to ubiquitination and protein repair as a specialized adaptation to persistent high hydrostatic pressure [26,83]. Similarly, the enrichment of DNA repair mechanisms in *B. nikolskii* parallels genomic studies of deep-water *A. affinis* and hadal fish and amphipods, which show positive selection in genes related to DNA repair and genetic information processing [23,84]. This collective evidence underscores that maintaining genomic and proteomic fidelity is fundamental to deep-water adaptation, further supported by the observation of lower mutation rates but elevated protein evolution rates (Ka/Ks) in deep-water teleosts globally [51].

These patterns find compelling parallels in other freshwater systems undergoing adaptive radiation along the benthic–pelagic axis. A striking and direct parallel is found in Arctic charr *Salvelinus alpinus* (Linnaeus, 1758), where a genetically distinct deep-water morph shows genomic signatures of selection on pathways for DNA repair, cardiac function, and neural synapse assembly, underscoring universal molecular challenges of deep freshwater colonization [21,85]. Similarly, studies on sympatric pairs of whitefish (*Coregonus* spp.) in postglacial lakes, which have independently diverged into benthic and pelagic ecomorphs, reveal a remarkable convergence in transcriptomic adaptations with Baikal sculpins. Pelagic whitefish ecotypes consistently upregulate genes associated with enhanced activity, including energy metabolism (e.g., oxidative phosphorylation genes), muscle contraction (e.g., specific parvalbumin variants), and lipid metabolism, reflecting a transcriptomic trade-off favoring survival and locomotion over growth [86,87,88,89]. In contrast, benthic forms upregulate genes related to protein synthesis, cell cycle, and growth [87]. This mirrors our findings where the pelagic *Comephorus* emphasizes metabolic and contractile machinery, while benthic *Batrachocottus* shows enrichment in ribosomal proteins and proteostatic pathways. The parallelism extends to the Baikal ecosystem itself. In particular, comparative transcriptomics of the sympatric benthic Baikal whitefish *Coregonus baicalensis* (Dybowski, 1874) and pelagic Baikal omul *Coregonus migratorius* (Georgi, 1775) revealed a similar dichotomy, with pelagic omul overexpressing metabolism genes (e.g., cytochrome c oxidase subunits) and benthic whitefish overexpressing protein synthesis and regulatory genes [90]. This pattern of benthic–pelagic specialization is a recurring theme in the broader Baikal sculpin radiation, where genomic studies indicate that diversification within genera like *Cottocomephorus* is driven by adaptation along this fundamental ecological gradient [89].

Moreover, the principles of adaptive genetic remodeling extend beyond depth-related pressures to other major aquatic transitions, highlighting convergent functional targets. For instance, genomic analyses of freshwater-adapted ecotypes in Pacific herring *Clupea pallasii* (Valenciennes, 1847) and diadromous sculpins, such as roughskin sculpin *Trachidermus fasciatus* (Heckel, 1837), reveal that adaptation to novel osmotic environments repeatedly targets pathways for osmoregulation, cellular homeostasis, and stress response, functional categories analogous to those under selection in deep-water adaptation [91,92]. Large-scale comparative genomic analyses further support that adaptation to novel ecological niches, whether along salinity, depth, or habitat gradients, consistently reshapes a core set of physiological systems, including ion transport, metabolism, sensory perception, and cellular integrity, despite species-specific genetic variations [93]. This is exemplified in marine threadfin breams (*Nemipterus* spp.), where deeper-dwelling species show positive selection in genes for osmoregulation, thermal response, energy metabolism, and skeletal integrity, highlighting a convergent molecular toolkit for deep-water life across phylogenetically distant fish [94].

In summary, the molecular adaptations of Baikal sculpins are not isolated phenomena but part of a convergent evolutionary narrative spanning both marine and freshwater deep-water environments. The patterns observed in cytoskeletal stability, lipid metabolism, proteostasis, and stress response align closely with findings from other species. Critically, comparisons with other freshwater radiations demonstrate that the ecological axis “benthic versus pelagic” is a powerful driver of divergent selection, leading to functional transcriptomic outcomes, even when the underlying genomic architecture may differ [21,88]. This underscores the paramount importance of ecological niche in shaping adaptive molecular evolution. Future work should also clarify the role of intersexual differences and ontogenetic changes in the expression of key genes in unique freshwater Baikal sculpins. This is particularly relevant for species whose pelagic larvae transition to a benthic lifestyle upon maturation. Furthermore, integrating transcriptomic data with proteomic, metabolomic, and epigenetic analyses will be crucial for building a comprehensive, mechanistic model of adaptation. Understanding these adaptations will ultimately reveal the limits of stability in living systems and elucidate how these unique species have evolved to not only survive but thrive in their deep-water environment.

## 5. Conclusions

The transcriptomic signatures of Lake Baikal sculpins demonstrate a range of convergent molecular adaptations, driven by distinct ecological pressures and evolutionary diversification. The transcriptomic divergence between the pelagic *Comephorus* and the benthic *Batrachocottus* species reflects fundamental ecological zonation, as well as fine-scale specializations within deep-benthic micro-niches. As secondary deep-water colonists with benthic ancestry, the pelagic *Comephorus* have undergone a radical evolutionary transformation. This is demonstrated by the upregulation of cytoskeletal, sarcomeric, and calcium-handling genes, which create a musculoskeletal system capable of sustained locomotion in a three-dimensional, high-pressure environment. In contrast, the benthic-abyssal specialists of both *Batrachocottus* species represent an alternative adaptive strategy, that prioritizes metabolic flexibility and strengthens cellular integrity through enhanced proteasome activity, ubiquitination, and DNA repair pathways. The transcriptomic divergences we identified not only reflect the specific ecological specializations of these Baikal sculpin species but also closely align with convergent adaptive strategies observed in marine and freshwater deep-water organisms. The focus on biomolecular stability, consistent with findings in hadal organisms, highlights that deep-water adaptation relies on systemic reinforcement of core molecular and cellular processes.

## Figures and Tables

**Table 1:** Summary of trawling conducted.

Number	Coordinates ^1^	Depth, m	Speed, Knots	Sculpin Species
1	53°56′824″ N 109°08′667″ E 53°59′156″ N 109°09′770″ E	795–810	2.4–2.2	*B. nikolskii*
2	53°59′231″ N 109°10′640″ E 53°56′326″ N 109°09′217″ E	795–805	2.8–2.1	*B. nikolskii*, *B. multiradiatus*
3	53°59′106″ N 109°07′275″ E 53°56′272″ N 109°06′882″ E	795–825	2.1–1.8	*B. nikolskii*
4	53°53′346″ N 109°06′222″ E 53°51′613″ N 109°08′093″ E	479–611	2.5–2.1	*B. multiradiatus*, *C. baicalensis*
5	53°51′357″ N 109°06′672″ E 53°53′445″ N 109°03′280″ E	342–385	2.5–1.9	*C. baicalensis*, *C. dybowski*
6	55°19′084″ N 109°41′746″ E 55°21′485″ N 109°39′743″ E	621–700	3.2–2.0	*C. baicalensis*, *C. dybowski*
7	52°09′931″ N 105°47′622″ E 52°13′311″ N 105°47′852″ E	986–1029	2.3–1.8	*C. baicalensis*, *C. dybowski*
8	52°11′953″ N 105°48′013″ E 52°08′510″ N 105°44′125″ E	1014–1070	2.2–2.1	*C. baicalensis*, *B. nikolskii*
9	52°29′034″ N 106°05′426″ E 52°31′383″ N 106°11′153″ E	457–560	3.6–2.7	*C. baicalensis*, *C. dybowski*
10	53°57′519″ N 109°10′823″ E 54°00′282″ N 109°11′063″ E	785–805	2.8–2.4	*B. nikolskii*, *B. multiradiatus*
11	54°00′124″ N 109°07′884″ E 53°57′822″ N 109°06′908″ E	805–825	2.4–2.1	*B. nikolskii*, *C. baicalensis*, *C. dybowski*
12	55°31′406″ N 109°43′917″ E 55°29′758″ N 109°46′528″ E	450–510	2.7–2.1	*B. multiradiatus*
13	55°27′130″ N 109°13′534″ E 55°21′485″ N 109°39′743″ E	218–267	2.6–1.9	*B. multiradiatus*
14	52°45′026″ N 106°46′342″ E 52°43′455″ N 106°48′789″ E	1214–1222	2.5–1.9	*C. baicalensis*, *B. nikolskii*
15	52°51′521″ N 106°57′913″ E 52°54′736″ N 106°55′270″ E	1370–1397	2.0–1.7	*C. baicalensis*

^1^ The coordinates indicated correspond to the start and end of the trawling.

**Table 2:** Characteristics of Baikal sculpins used in the study.

Species	Average Length, TL, cm	Weight, g	Specimens Number
*C. baicalensis*	16.17 ± 3.42	25.57 ± 15.38	23
*C. dybowski*	12.43 ± 1.19	10.38 ± 3.43	21
*B. multiradiatus*	14.99 ± 3.09	51.18 ± 29.24	18
*B. nikolskii*	20.45 ± 2.96	141.02 ± 47.38	22

**Table 3:** Primers designed for qPCR analysis.

Name	Primer Sequence (5′–3′)	Product Size (bp)
*RAG1* F	GGAGACCCAGACAACGATGG	113
*RAG1* R	CGGCTGGGTTTGACCTTTTG	
*LDHA* F	AGCCTTTGAGCTCAGCATGT	186
*LDHA* R	AACACTGTTCACCCGGTTCA	
*TNNT3* F	TATAGTGACACGCGATCCGC	122
*TNNT3* R	CCTTATTGAGTGCCAGCGGA	
*PIP4K2C* F	AGCCCTCTCCCCGATTATTCT	206
*PIP4K2C* R	CGTAACCCCTCCAGTGCTTT	
*PDCL3* F	GTCCAGGAGTCCTCAGCATG	120
*PDCL3* R	GAAAGGACAGCGACTCGGAG	
*ECE1* F	TGGGCTACATGATGGCCAAG	254
*ECE1* R	TGTTGCCTCCTCTATGGGGA	
*GNAI* F	GTCACCCGCTGGACAGTTTA	141
*GNAI* R	TGGCTGACCCAATCACAGAA	
*ACTG1* F	ATTAGGATGCTGACAGGCCG	163
*ACTG1* R	GGGAGGTCAAAGCGACAGTT	
*MDH1* F	GTCTAGTCCGACTCTGAGCAG	128
*MDH1* R	TACAGCAGGGAGTAGGCGAT	
*FABP3* F	GTAGCTCTCACTGATCCGCC	102
*FABP3* R	GCCCCTGGCAACACTACTTA	
*RTN4L* F	AGAGCGTTGCATGATGGGAA	136
*RTN4L* R	CCAGACAGAGTTGCAGAGCA	
*HBAX* F	CCCAGGATGAGCAGCATGAA	126
*HBAX* R	CTGGCAGAGAGATACCGCTG	
*APOEb* F	AAGGACATGCTGGATGCCAA	115
*APOEb* R	GCTTGTGTGAATAGGTGCCG	
*KRT50* F	TTCATGCTGAGCTGGGACTG	150
*KRT50* R	GGAAGTCTGGTTCCAGAGCA	
*COX5A* F	ATCTTTCTCCCTCTCCGCCT	145
*COX5A* R	GAGCTCTGCAGCGTTTTGAG	
*MT-CYB* F	ACCTCTTAGGAGACCCGGAC	137
*MT-CYB* R	GGCTAGAACGCCTCCAAGTT	
*NID2* F	AACACTGCTGACCCTCCATG	148
*NID2* R	AGGCGTGACACATAACAGCA	

**Table 4:** Sequencing data quality statistics.

Sample	Raw_Bases	Raw_Reads	Clean_Reads	Error_Rate	Q20	Q30	GC_pct
*C. baicalensis*	18.08G	120,554,062	119,466,812	0.01	99.05	96.18	53.06
*C. dybowski*	18.36G	122,419,718	121,200,064	0.01	99.08	96.28	53.03
*B. multiradiatus*	19.33G	128,862,796	127,653,460	0.01	99.13	96.31	53.13
*B. nikolskii*	18.31G	122,040,414	120,783,398	0.01	99.21	96.58	52.67

## Data Availability

The raw sequence data have been submitted to the Gene Expression Omnibus (NCBI-GEO) repository (accession number: GSE308109, https://www.ncbi.nlm.nih.gov/geo/query/acc.cgi?acc=GSE308109, accessed on 26 November 2025).

## Supplementary Materials

The following supporting information can be downloaded at: https://www.mdpi.com/article/10.3390/biology14121762/s1, S1: Transcriptome assembly report; S2: Trinity gene counts; S3: Annotation of all DEGs without a threshold; S4: Annotation of DEGs with a significance threshold set at *p* = 0.001 (logFC = 2); S5: DEG counts, logCPM; S6: DEG counts, logFC.
