# Supplementary material for: Comparative Transcriptomics as a Key to Understanding the Adaptation Mechanisms of Baikal Sculpins to the Deep-Water Habitat
Source: Biology (Basel). 2025 Dec 9;14(12):1762. doi: 10.3390/biology14121762 (PMC12730502; doi:10.3390/biology14121762)
Supplement: Supplementary file 1 [file biology-14-01762-s001.zip › S1_Transcriptome assembly_report.pdf]

Report

	GSE308109_Trinity
# contigs (>= 0 bp)	152088
# contigs (>= 1000 bp)	48212
# contigs (>= 5000 bp)	226
# contigs (>= 10000 bp)	1
# contigs (>= 25000 bp)	0
# contigs (>= 50000 bp)	0
Total length (>= 0 bp)	143410159
Total length (>= 1000 bp)	88044478
Total length (>= 5000 bp)	1300531
Total length (>= 10000 bp)	11944
Total length (>= 25000 bp)	0
Total length (>= 50000 bp)	0
# contigs	96763
Largest contig	11944
Total length	121871112
Reference length	6430384
N50	1548
N90	646
auN	1744.0
L50	26484
L90	75250
# misassemblies	0
# misassembled contigs	0
Misassembled contigs length	0
# local misassemblies	0
# scaffold gap ext. mis.	0
# scaffold gap loc. mis.	0
# unaligned mis. contigs	3
# unaligned contigs	96699 + 20 part
Unaligned length	121828148
Genome fraction (%)	0.336
Duplication ratio	6.433
# N's per 100 kbp	0.00
# mismatches per 100 kbp	21048.32
# indels per 100 kbp	4196.69
Largest alignment	1453
Total aligned length	29285
NA50	-
NA90	-
auNA	0.2
LA50	-
LA90	-

All statistics are based on contigs of size >= 500 bp, unless otherwise noted (e.g., "# contigs (>= 0 bp)" and "Total length (>= 0 bp)" include all contigs).

Misassemblies report

	GSE308109_Trinity
# misassemblies	0
# contig misassemblies	0
# c. relocations	0
# c. translocations	0
# c. inversions	0
# c. interspecies translocations	0
# scaffold misassemblies	0
# s. relocations	0
# s. translocations	0
# s. inversions	0
# s. interspecies translocations	0
# misassembled contigs	0
Misassembled contigs length	0
# possibly misassembled contigs	19
# possible misassemblies	27
# local misassemblies	0
# scaffold gap ext. mis.	0
# scaffold gap loc. mis.	0
# unaligned mis. contigs	3
# mismatches	6164
# indels	1229
# indels (<= 5 bp)	1179
# indels (> 5 bp)	50
Indels length	2270

All statistics are based on contigs of size >= 500 bp, unless otherwise noted (e.g., "# contigs (>= 0 bp)" and "Total length (>= 0 bp)" include all contigs).

Unaligned report

	GSE308109_Trinity
# fully unaligned contigs	96699
Fully unaligned length	121798321
# partially unaligned contigs	20
Partially unaligned length	29827
# N's	0

All statistics are based on contigs of size >= 500 bp, unless otherwise noted (e.g., "# contigs (>= 0 bp)" and "Total length (>= 0 bp)" include all contigs).

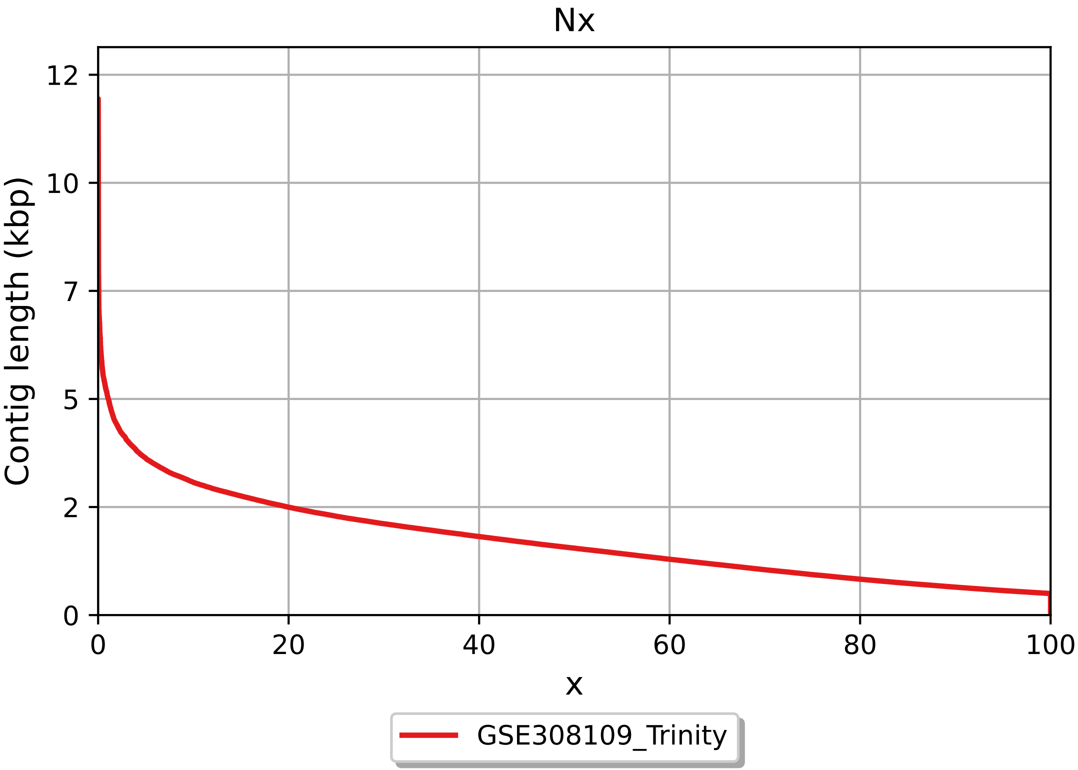

Nx
Contig length (kbp)
12
10
7
5
2
0
0
20
40
60
80
100
x
GSE308109_Trinity

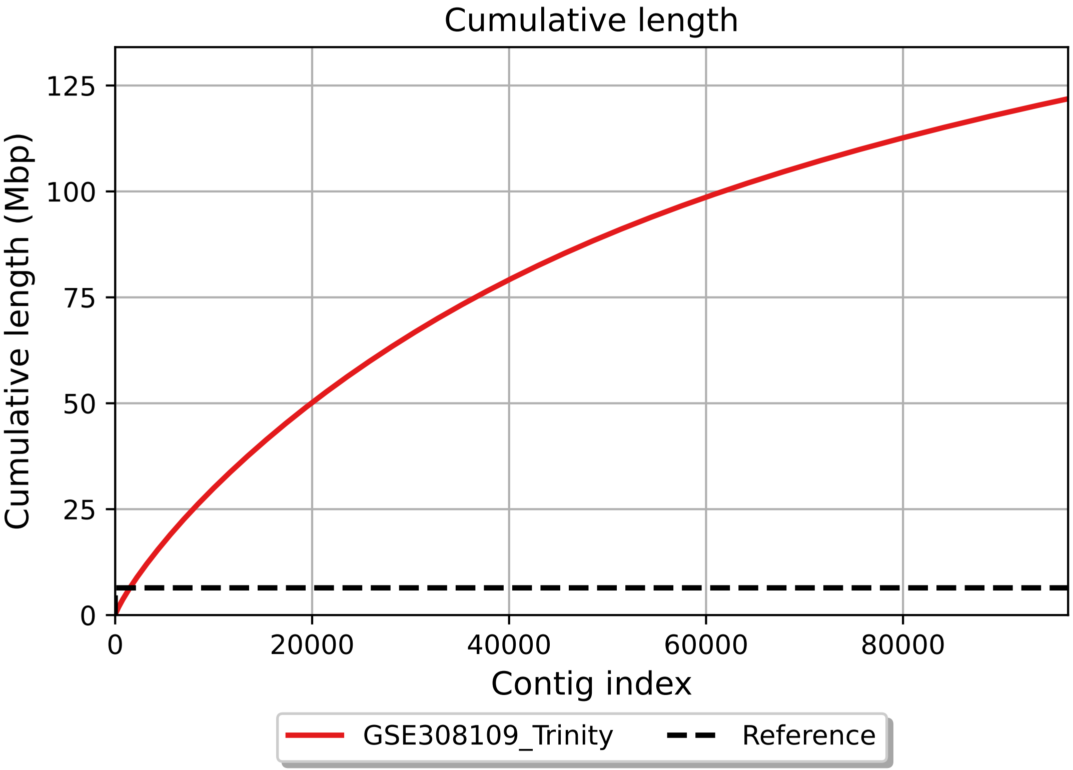

Cumulative length
Cumulative length (Mbp)
0
25
50
75
100
125
0
20000
40000
60000
80000
Contig index
GSE308109_Trinity
Reference

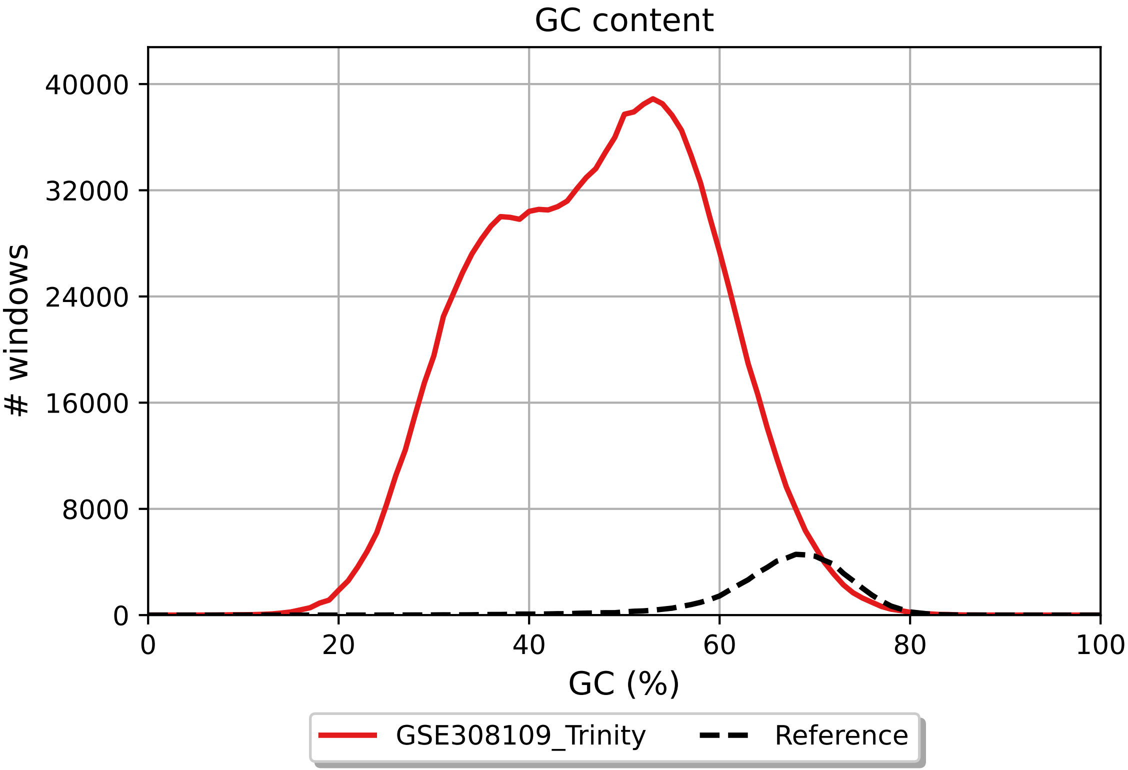

GC content
windows
40000
32000
24000
16000
8000
0
0
20
40
60
80
100
GC (%)
GSE308109_Trinity
Reference

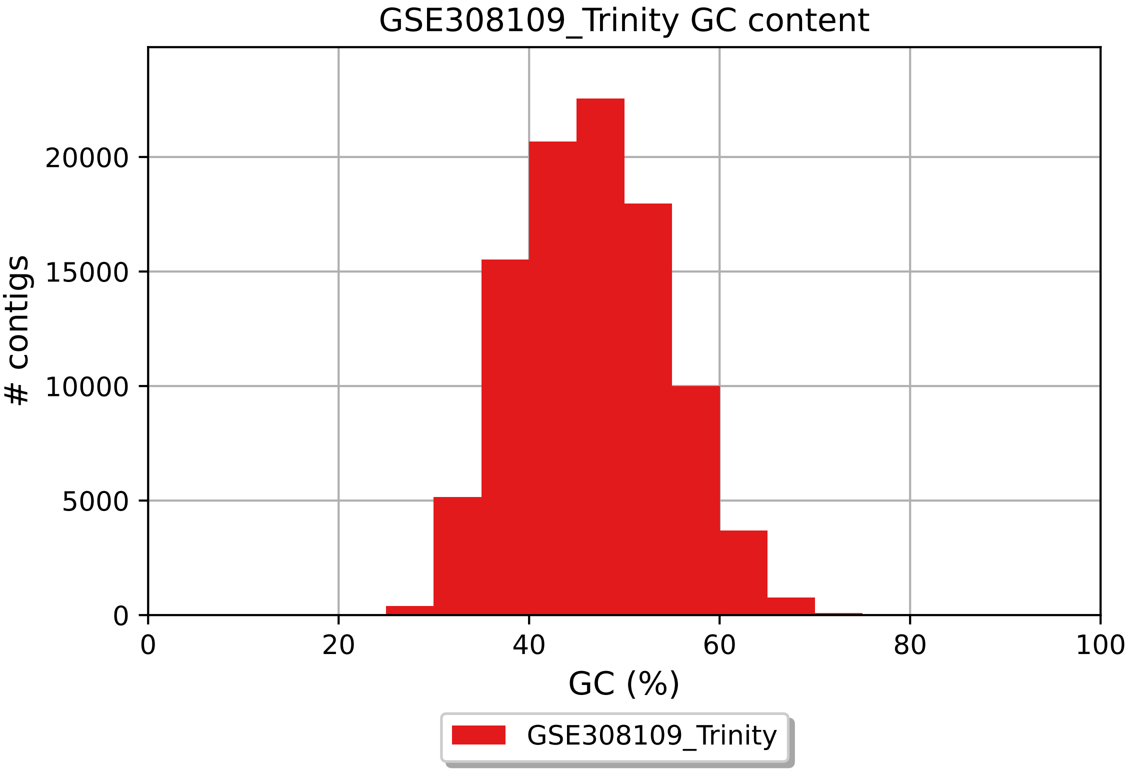
GSE308109_Trinity GC content
contigs
0
5000
10000
15000
20000
0
20
40
60
80
100
GC (%)
GSE308109_Trinity

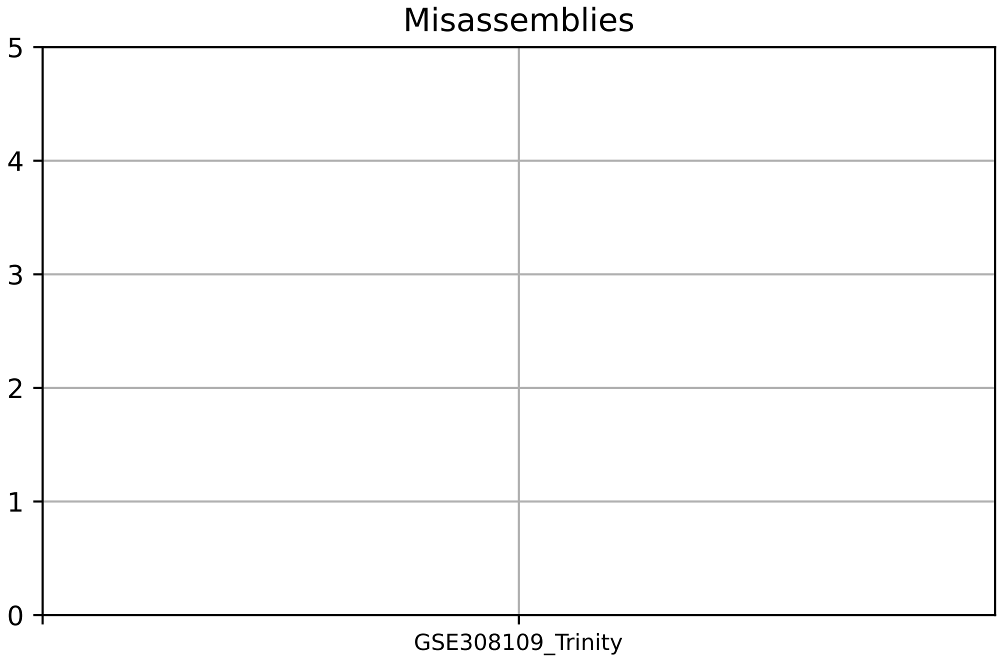
Misassemblies
5
4
3
2
1
0
GSE308109_Trinity

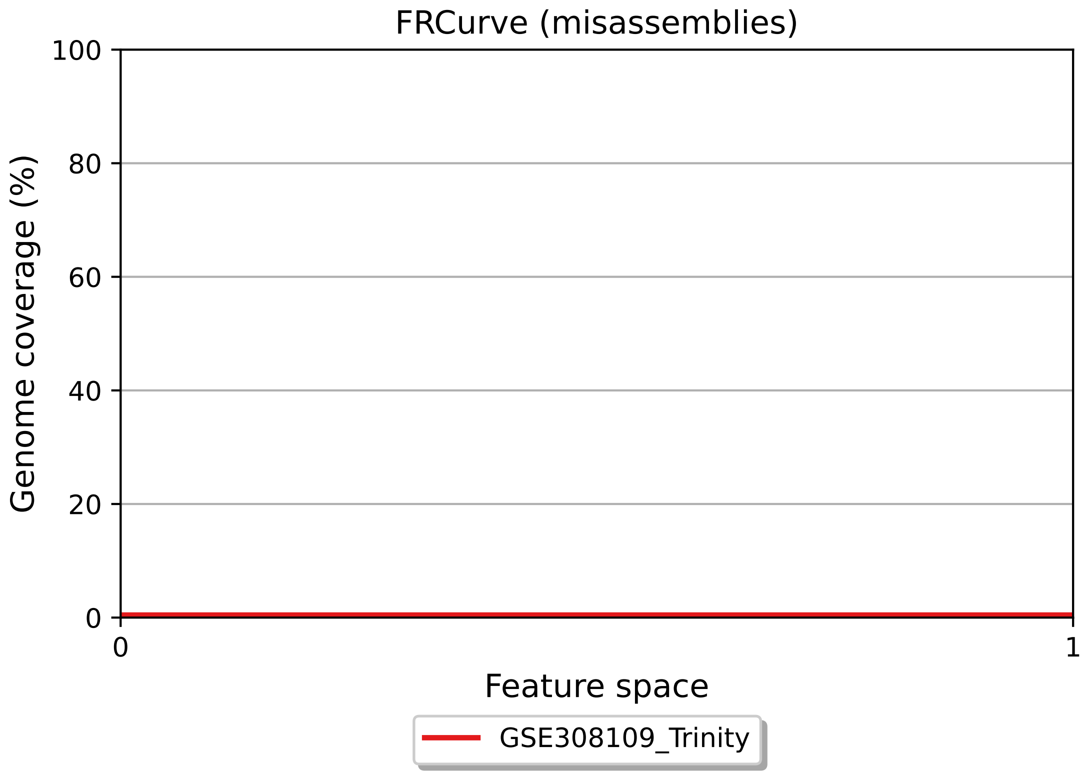

FRCurve (misassemblies)
100
80
60
40
20
0
Genome coverage (%)
0
1
Feature space
GSE308109_Trinity

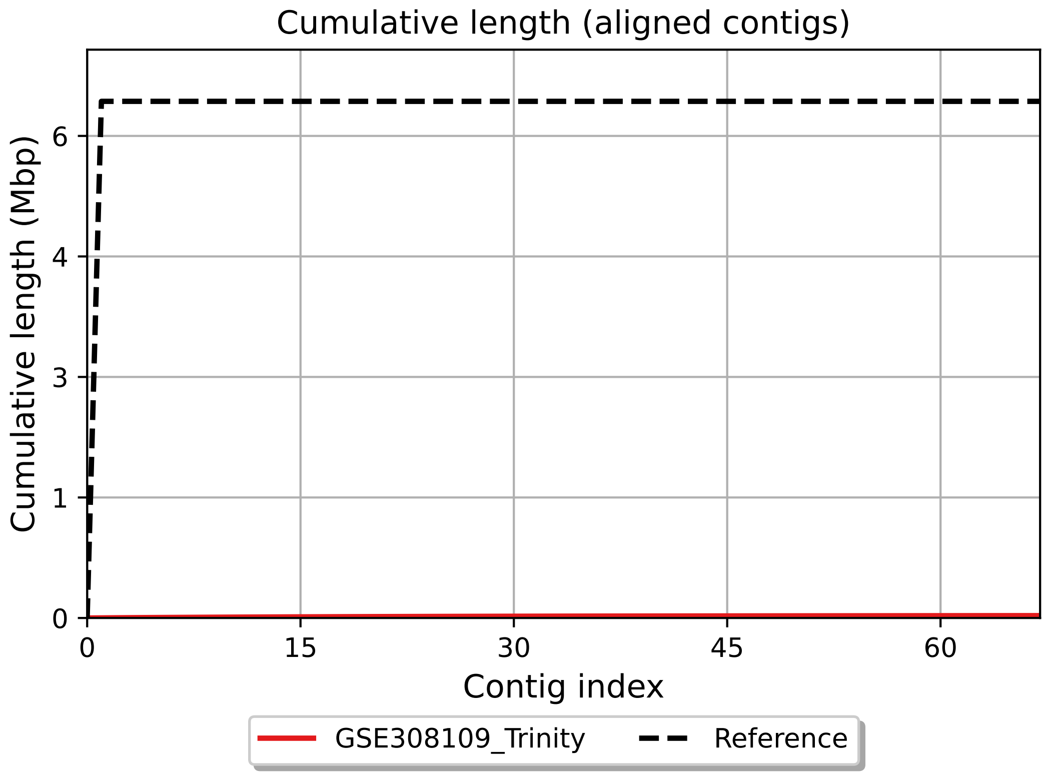
Cumulative length (aligned contigs)
Cumulative length (Mbp)
0
1
3
4
6
0
15
30
45
60
Contig index
GSE308109_Trinity
Reference

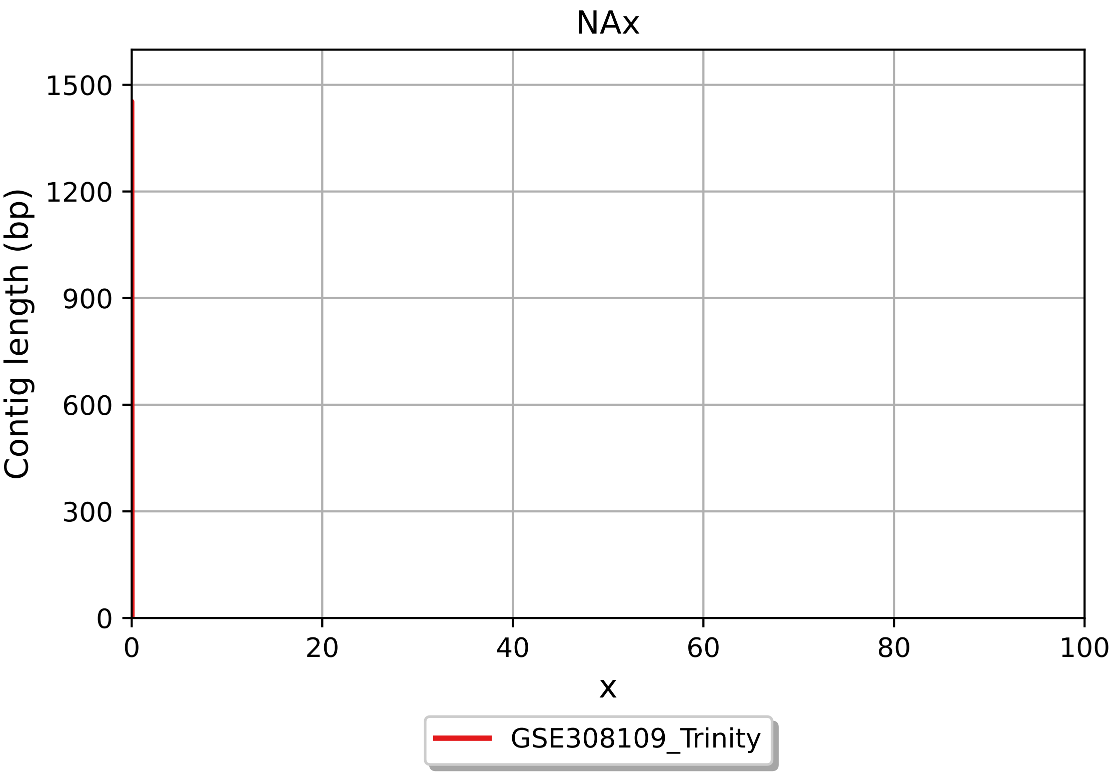

NAx
Contig length (bp)
0
300
600
900
1200
1500
0
20
40
60
80
100
x
GSE308109_Trinity
